# CGG repeats trigger translational frameshifts that generate aggregation-prone chimeric proteins

**DOI:** 10.1093/nar/gkac626

**Published:** 2022-07-29

**Authors:** Shannon E Wright, Caitlin M Rodriguez, Jeremy Monroe, Jiazheng Xing, Amy Krans, Brittany N Flores, Venkatesha Barsur, Magdalena I Ivanova, Kristin S Koutmou, Sami J Barmada, Peter K Todd

**Affiliations:** Department of Neurology, University of Michigan, Ann Arbor, MI 48109, USA; Neuroscience Graduate Program, University of Michigan, Ann Arbor, MI 48109, USA; Department of Neurology, University of Michigan, Ann Arbor, MI 48109, USA; Neuroscience Graduate Program, University of Michigan, Ann Arbor, MI 48109, USA; Department of Genetics, Stanford University School of Medicine, Stanford, CA 84305, USA; Department of Chemistry, University of Michigan, Ann Arbor, MI 48109, USA; Department of Neurology, University of Michigan, Ann Arbor, MI 48109, USA; Department of Neurology, University of Michigan, Ann Arbor, MI 48109, USA; VA Ann Arbor Healthcare System, Ann Arbor, MI 48105, USA; Department of Neurology, University of Michigan, Ann Arbor, MI 48109, USA; Cellular and Molecular Biology Graduate Program, University of Michigan, Ann Arbor, MI 48109, USA; Department of Pathology, University of Michigan, Ann Arbor, MI 48109, USA; Department of Neurology, University of Michigan, Ann Arbor, MI 48109, USA; Biophysics Program, University of Michigan, Ann Arbor, MI 48109, USA; Department of Chemistry, University of Michigan, Ann Arbor, MI 48109, USA; Department of Neurology, University of Michigan, Ann Arbor, MI 48109, USA; Department of Neurology, University of Michigan, Ann Arbor, MI 48109, USA; VA Ann Arbor Healthcare System, Ann Arbor, MI 48105, USA

## Abstract

CGG repeat expansions in the *FMR1* 5’UTR cause the neurodegenerative disease Fragile X-associated tremor/ataxia syndrome (FXTAS). These repeats form stable RNA secondary structures that support aberrant translation in the absence of an AUG start codon (RAN translation), producing aggregate-prone peptides that accumulate within intranuclear neuronal inclusions and contribute to neurotoxicity. Here, we show that the most abundant RAN translation product, FMRpolyG, is markedly less toxic when generated from a construct with a non-repetitive alternating codon sequence in place of the CGG repeat. While exploring the mechanism of this differential toxicity, we observed a +1 translational frameshift within the CGG repeat from the arginine to glycine reading frame. Frameshifts occurred within the first few translated repeats and were triggered predominantly by RNA sequence and structural features. Short chimeric R/G peptides form aggregates distinct from those formed by either pure arginine or glycine, and these chimeras induce toxicity in cultured rodent neurons. Together, this work suggests that CGG repeats support translational frameshifting and that chimeric RAN translated peptides may contribute to CGG repeat-associated toxicity in FXTAS and related disorders.

## INTRODUCTION

Short tandem repeat expansions cause over 50 human neurological disorders with no known cure (1). CGG trinucleotide repeat expansions have recently emerged as a major cause of multiple neurological conditions, including neuronal intranuclear inclusion disease (NIID) (2,3), oculopharyngodistal myopathy (OPDM) and leukodystrophy (OPML) (2,4–6), and adult onset leukoencephalopathy (7). The best studied CGG repeat expansion resides in the 5’ untranslated region (UTR) of the *FMR1* gene (8). Very large expansions (>200 repeats) trigger Fragile X Syndrome through repeat methylation, epigenetic changes, and *FMR1* transcriptional silencing (9). However, more intermediate sized expansions (55–200 CGG repeats) at this locus cause Fragile X-associated tremor/ataxia syndrome (FXTAS), an adult-onset neurodegenerative disorder that affects upwards of one in 5000 men. The pathological hallmark of FXTAS is ubiquitin and p62 positive intranuclear neuronal inclusions found throughout the cerebrum and cerebellum and associated with widespread neuronal loss (10–13).

There are two non-exclusive models for how CGG repeat expansions might elicit a gain-of-function toxicity. First, CGG repeats may be toxic as mRNA, either by binding to and sequestering RNA-binding proteins or by repeat RNA gelation into phase separated nuclear droplets (1,14–19). Second, CGG repeats support translation initiation in the absence of an AUG start codon via a process known as repeat-associated non-AUG (RAN) translation (20–25). At CGG repeats, RAN translation occurs in all three potential reading frames at different efficiencies and with most initiation beginning from specific near-AUG cognate codons just 5’ to the repeat element (21,22).

The most abundant product generated by RAN translation from CGG repeats is FMRpolyG (22), a polyglycine containing peptide that accumulates within neuronal inclusions in both patients and model systems (21,26,27). Removal of the near-AUG cognate codons that support FMRpolyG production from the *FMR1* 5’UTR markedly limits the ability of CGG repeats to trigger inclusion formation and induce toxicity in multiple model systems, including flies, human cells and transgenic mouse models (21,27–30). However, studies to date have not directly assessed whether FMRpolyG production in isolation is sufficient to recapitulate the FXTAS relevant phenotypes in neurons. In particular, the potential toxic contributions of products generated via RAN translation in other reading frames, including polyarginine and polyalanine products have not been explored. With other repeat expansions, constructs that express each RAN peptide in the absence of the repeat element (via an AUG initiation site and degenerate codon sequences) have been very informative in discerning which RAN product is most intrinsically toxic (29,31,32). To this end, we utilized a similar strategy to study the impact of CGG RAN translation products on toxicity in neurons.

Here, we show that expression of FMRpolyG from an AUG initiated construct is markedly less toxic and less likely to form inclusions in the absence of the CGG repeat. To explore why this might occur, we evaluated whether removal of near-cognate initiation codons in one reading frame influenced translation in other reading frames. Surprisingly, mutating the upstream polyR-frame RAN initiation site increased polyG-frame translation. This cross-reading frame effect is explained by an R-to-G (+1) translational frameshift that occurs within the CGG repeat after incorporating 1–4 arginines. Frameshifting at CGG repeats is dependent on RNA sequence and 3’ structural elements within *in vitro* assays but is largely confined to the R-to-G reading frame in cells and neurons, suggesting an additional impact of polyarginine translation *in vivo*. Products that mimic this chimeric frameshifted protein exhibited distinct biophysical properties and greater neuronal toxicity than polyR or polyG peptides alone. Together, this data provides evidence that frameshifting at CGG repeats produces chimeric frameshift products that may contribute to neurodegeneration.

## MATERIALS AND METHODS

### Plasmid construction

The relevant sequences of all reporters used in this study are included in Supplemental Table 1.

GFP-pGW was modified to include restriction sites PmeI and XmaI. The AUG of GFP was mutated to a GGG (nGFP-pGW). AUG-V5 polyR-CGG_100_ and AUG-V5 polyA-CGG_100_ in pUAST were digested with HpaI and AgeI. The DNA fragment was inserted into the modified nGFP-pGW using PmeI and XmaI. AUG-V5 polyG-CGG_100_ was inserted into FMRpolyG nGFP pGW using HindIII and XhoI. Gene blocks containing the ATG V5 FMRpolyG sequence upstream of an optimized GGN (mixture of GGC, GGG, GGA and GGT) sequence with flanking HindIII and PmeI restrictions sites, or the ATG V5 FMRpolyR sequence upstream of an optimized CGN (mixture of AGA, AGG, CGC, CGG, CGA and CGT) sequences with flanking HindIII and AvrII restriction sites were ordered and then inserted into nGFP pGW via restriction digest.

FMRpolyG-nGFP pGW (CGG_115_) plasmids with polyR-frame ACG mutations were generated by inserting gene blocks containing the native 5’UTR with the desired polyR-initiation site mutation and an EarI site in place of the CGG repeat upstream of the nGFP sequence in an empty vector (with no other EarI sites) using HindIII and AvrII restriction sites. CGG_115_ fragment isolated by EarI digest from a donor plasmid was then inserted into the EarI site within the *FMR1* 5’UTR. Gene blocks of R50G50, and R4G96 optimized codon sequences flanked by HindIII and AvrII restriction sites were inserted into nGFP-pGW vectors by restriction digest at HindIII and AvrII.

pcDNA3.1(+) vectors expressing CGG_n_-polyG-NL3xFLAG, and firefly luciferase were described previously (22,33). Dual-tagged reports were generated using short DNA oligos creating an AUG-V5 tag, one tag for each reading frame, flanked by EcoRI and NarI. The tags for the different reading frames were then inserted upstream of the CGG_n_-polyR-NL3xFLAG, CGG_n_-polyG-NL3xFLAG, and CGG_n_-polyA-NL3xFLAG vectors, resulting in nine different vectors.

### *In vitro* transcription

RNA used for transfection into HEK293Ts was generated from pcDNA3.1(+) vectors linearized with PspOMI. Functionally capped RNA was synthesized using the Hiscribe T7 ARCA mRNA kit (with tailing) (New England Biolabs) according to manufacturer's instructions.

### *In vitro* translation assay

The mRNA was generated from T7 polymerase transcription reactions of DNA oligonucleotides and purified via urea-PAGE (34). Transcribed RNA sequences are shown in Supplemental Table 2. Bulk *Escherichia coli* tRNA was purchased from Sigma and amino acylated with S100 extract (35,36).

*Escherichia coli* MRE600 tight coupled 70S ribosome were prepared as previously described (36). Initiation complexes (ICs) with all mRNAs of interest were prepared in 1 × 219–Tris Buffer (50 mM Tris pH 7.5, 70 mM NH_4_Cl, 30 mM KCl, 7 mM MgCl_2_, 5 mM β-ME) with 1 mM GTP and as previously published (36,37).

Ternary complexes (TCs; total aa-tRNA:EF-Tu:EF-G) were formed as previously published (37). Amino acid addition reactions were conducted by mixing TCs (10 μM total aa-tRNA, 20 μM EF-Tu, 10 μM EF-G) and ICs (100 nM) at 37°C. Reaction was quenched with 2 μl of 1M KOH. Reaction products were separated via electrophoretic TLC, 1200 V for 25 min. eTLCs were visualized by phosophorimaging and quantified with ImageQuant software (36).

### Primary neuron culture, transfection and longitudinal imaging

Primary cortical neurons were dissected from embryonic day 20 rat pups and plated at 0.6 × 10^6^ cells/ml, as previously described (38–40). Neurons were transfected 4 days after plating with pGW1-mApple alone (for peptide toxicity assays) or with indicated RAN-expressing nGFP constructs using Lipofectamine 2000 (ThermoFisher). pGW1-mApple is expressed throughout the neuron and is used in downstream analysis to track neuronal death across imaging timepoints.

For peptide toxicity assays, fresh peptides were dissolved in 0.1 M NaCl, 25 mM sodium phosphate pH 7.4, filter sterilized, and added to a final concentration of 0.1 mM immediately following transfection with pGW1-mApple. Addition of fibrils of α-synuclein (0.1 mM) were used as a positive control. For fibril polymerization, recombinantly expressed full-length α-synuclein was purified as previously described. α-Synuclein (100 μM in 150 mM NaCl, 25 mM Na phosphate pH 7.5, 1 mM Na azide and 10 μM ThioflavinT (ThT)) was polymerized in 384-well glass bottom plates (Greinier, 781892) by shaking (200 rpm) at 37°C using FLUOstar Omega (BMG Labtech Inc.). The progress of reaction was monitored by measuring ThT fluorescence at 440 nm (excitation) and 490 nm (emission). Samples were analyzed for fibril formation by TEM. The polymerization buffer was exchanged by spinning α-synuclein fibrils for 15 min at 17 000 × g. The pellet was resuspended with 1× PBS. Fibrils were sonicated in a Diagenode Bioruptor device for 15 cycles (30 s ON and 30 s OFF each), followed by concentration determination of α-synuclein (monomer) at 280 nm with an extinction coefficient of 5960 M^−1^ cm^−1^. Prior absorbance measurements, fibrils were denatured by mixing 1 μl with 4 μl 6M Guanidine HCl, and the concentration of sonicated fibrils was adjusted to 30 μM (monomer).

Longitudinal microscopy was performed as previously described (38–40), beginning 24 h after transfection, and at 24 h intervals for a total of 10 imaging timepoints. Neurons did not receive media changes throughout imaging. Image processing and survival analysis were performed by an original code written in Python or ImageJ macro language. Cumulative risk of death was determined using cox proportional hazards analysis performed with the publicly available R survival package.

### Semi-automated inclusion analysis

Processed GFP images obtained during survival analysis were manually reviewed to define a subset of ROIs (*n* = 50/group) as ‘aggregated’ or ‘diffuse’. We then determined the coefficient of variation (CV) for the GFP signal in these ROIs. CV, the ratio of the standard deviation to the mean GFP intensity, is proportional to the spatial variability of fluorescence intensity within an ROI, and has been previously used to identify puncta in this unbiased and high-throughput system (41,42). The use of CV to detect aggregates was validated by creating a receiver-operator characteristic (ROC) curve, demonstrating that a CV threshold of 1.0 differentiates between ROIs with aggregates and those with diffuse GFP signal with both high sensitivity (95.9%) and high specificity (93.8%). This CV threshold was then applied to the broad dataset to compare survival of neurons with aggregates to those without aggregates. Accuracy of these models is further confirmed by the limited erroneous detection of ‘aggregates’ in the nGFP control transfected cells.

### HEK293T cell culture

HEK293T cells (American Type Culture Collection (ATCC)) were maintained in Cytiva DMEM F12 1:1 media (Fisher), 10% fetal bovine serum (Bio-Techne) (v:v), an 1% penicillin-streptomycin (ThermoFisher) (v:v). Cells were passaged when ∼70–80% confluent with Trypsin-EDTA (0.25%), phenol red (ThermoFisher).

### Immunocytochemistry and confocal imaging

HEK293T cells were plated on 8-well glass chamber slides at 5 × 10^4^ cells/well and transfected 24 h later with 25 ng total DNA using Fugene HD (Promega) according to manufacturer instructions. Cells were washed with PBS containing 1 mM MgCl_2_ and 0.1 mM CaCl_2_ (PBS-MC) 24 h after transfection and fixed in 4% paraformaldehyde/4% sucrose in PBS-MC for 15 min at room temperature. Cells were permeabilized in 0.1% Triton-X (Sigma-Aldrich) in PBS-MC, and blocked in 2% BSA (Millipore Sigma) for 20 min. Cells were incubated in primary antibody for V5 (1:2000, mouse, Abcam, ab27671) and FLAG (1:1000, rabbit, Sigma, F7435) for 2 h at room temperature, washed 3X in PBS-MC, then incubated in 1:1000 goat anti-mouse IgG (H + L) highly cross-adsorbed secondary antibody, Alexa Fluor 488 (ThermoFisher, A11029) and goat anti-rabbit IgG (H + L) cross-adsorbed secondary antibody, Alexa Fluor 555 (ThermoFisher, A21428) for 1 h at room temperature. After incubation with secondary antibody, cells were washed 3× with PBS-MC, and coverslipped with ProLong Gold Antifade with DAPI (ThermoFisher). Confocal imaging was performed on an inverted Olympus FV1000, laser-scanning confocal microscope, using a 20×, 40× or 60× objective. Co-localization analysis was performed using the CoLoc2 Fiji plugin using default settings on background subtracted images. Pearson's correlation co-efficient describes how the intensity of the fluorescence in one channel changes with the fluorescence intensity in a second channel, while the Manders’ coefficient reflects the degree that the objects in two channels colocalize.

### Luciferase assay

HEK293T cells were plated on 96-well plates with 2 × 10^4^ cells/well, and co-transfected 24 h later with 5 ng nanoluciferase reporter and 5 ng pGL4.13 (firefly luciferase, FFLuc) using Fugene HD (Promega). Cells were lysed 24 h post-transfection in 60 ul Glo Lysis Buffer, 1× (Promega) for 15 min at room temperature. 25 ul lysate was transferred across two black well plates. 25 ul 1:50 Nano-Glo substrate:buffer (Promega) was added to one plate, and 25 ul ONE-Glo reagent (Promega) was added to the other for nLuc and FFLuc quantification, respectively. nLuc was normalized to FFLuc to control for variation in transfection efficiencies.

### Western blot and immunoprecipitation

HEK293T cells were plated on 12- or 6-well plates with 2 × 10^5^ or 4 × 10^5^ cells/well, respectively, and transfected with 100 or 200 ng nanoluciferase reporter using Fugene HD (Promega). 24 h post-transfection, cells were lysed in RIPA buffer supplemented with mini-complete protease inhibitors (Sigma) and boiled for 5 min at 90°C in 6× SDS for immediate immunoblotting on 12% SDS-PAGE gels. Lysates for immunoprecipitation were incubated with 20 or 40 μl FLAG (Sigma, M8823) or V5(ProteinTech, v5tmak-20)-conjugated beads on rotating bar at 4°C overnight. Lysate-bound beads were washed 3× with TBS before eluting off beads by boiling at 90°C in 2× Lammaeli buffer. Elutions were resolved on 12% SDS-PAGE gels. Gels were transferred for 16 h at 4°C at 45V onto PVDF membranes (2 μm, Bio-rad). Membranes were blocked in 5% milk in TBS-T for 1 h at room temperature, incubated in in primary antibody for V5 (1:2000, mouse, Abcam, ab27671) and FLAG (1:1000, mouse, Sigma, F1804), and tubulin (1:1000, mouse, DSHB, E7) for 2 h at room temperature or overnight at 4°C, washed 3× in TBS-T, then incubated in 1:5000 HRP-conjugated goat-anti-mouse (115-035-146) or goat-anti-rabbit (111-035-144) antibodies (Jackson ImmunoResearch Laboratories) for 1 h at room temperature. After secondary incubation, membranes were washed 3× with TBS-T, then incubated with 1:1 Western Lightning Plus-ECL (50-904-9326, Fisher) and developed.

### RNA quantification

HEK293T cells were plated on 12-well plates with 2 × 10^5^ cells/well and transfected with 100 ng plasmid. 24 h post-transfection, total RNA was collected using Quick-RNA Miniprep Kit (Zymo Research), followed by incubation with Turbo DNase (ThermoFisher) to eliminate DNA. cDNA was synthesized from 500 ng RNA/reaction with iScript cDNA synthesis kit (Bio-Rad). Quantitative real-time PCR reactions of equal amounts of cDNA were performed according to manufacturer instructions using TaqMan Fast Advance Master Mix (Applied Biosystems, 4444557) on an Applied Biosystems Quant Studio 3 machine for 40 cycles using fast cycling parameters (95°C for 20 s, 95°C for 1 s, 60°C for 20 s). All plates included a standard dilution curve representing 2× to 0.02× of the RNA concentration utilized for all primer sets (Supplemental Table 3) to ensure linearity. Equivalent efficiency of individual primer sets was confirmed prior to data analysis. All samples were run in triplicate. The level of mRNA of interest was normalized to GAPDH mRNA and expressed as the change in gene expression to samples with native sequences (i.e. wildtype).

### Liquid chromatography–mass spectrometry (LC–MS/MS)

HEK293T cells were plated on 15 cm plates with 3 × 10^6^ cells/well and transfected with 1.5 μg plasmid. Cells were lysed in RIPA buffer supplemented with mini-complete protease inhibitors (Sigma) and incubated with 100 μl FLAG (Sigma, M8823) or V5 (ProteinTech, v5tmak-20)-conjugated beads on rotating bar at 4°C overnight. Washed beads were digested with trypsin or Glu-C, for peptide identification at the University of Michigan Proteomics Resource Facility using a hybrid quadrupole-orbitrap mass spectrometer (Q Exactive HF, Thermo Scientific) coupled to nano-UHPLC (Ultimate 3000 RSLC Nano, ThermoScientific). Proteome Discoverer (v2.1; Thermo Fisher) was used for data analysis, using the SwissProt human protein database (42054 sequences). Percolator algorithm (PD2.1) was used to determine the false discovery rate (FDR) and protein/peptides with ≤1% FDR were retained for further analysis. MS/MS spectra assigned to the peptides of interest were manually examined.

### Circular dichroism

Lyophilized G6 and R6 (Genscript) were dissolved to 0.3 mg/ml with a CD buffer (0.1 M NaF 25 mM Na phosphate pH 7.5). Frozen stocks of R3G3 (10–12.5 mg/ml in water, Genscript) were thawed on ice, and diluted to 1.2 mg/ml with water. Stocks of NaF and Na phosphate pH7.5 were added to match the composition of the CD buffer. Then R3G3 solutions were additionally diluted to 0.3 mg/ml (final). R6/G6 mixtures (1:1) were prepared by mixing equivalent volumes of R6 and G6 (0.6 mg/ml in CD buffer) to a final 0.3 mg/ml concentration of each peptide. Peptide samples were incubated at 37°C without shaking for two weeks. Spectra were collected immediately (*T* = 0 day), 2, 4 and 14 days after sample preparation. CD spectra were collected from samples prepared by two to four independent experiments.

Data collection was performed using a Jasco J-1500 circular dichroism spectrophotometer. Samples were scanned from 190 to 260 nm with a scanning speed of 100 nm/min, data interval of 1 nm, and 5 nm bandwidth. Spectra are the average of 32 accumulations. All spectra were corrected for the background, by subtracting the buffer spectra using Jasco software, and baselined by setting the first value of 260 nm to zero.

### Transmission electron microscopy

TEM was performed as previously described (40). Briefly, R6 (0.6 mg/ml), G6 (0.6 mg/ml), mixture R6/G6 (total peptide concentration 0.6 mg/ml) and hybrid R3G3 (0.6 mg/ml) in 0.1M NaCl_2_ 5 mM Na phosphate pH 7.4 were aggregated by incubating at 37°C for 12 days. Negatively stained specimens for TEM were prepared by applying 5 μl sample to hydrophilic 400 mesh carbon-coated Formvar support films mounted on copper grids (Ted Pella, Inc., 01702-F). Samples were allowed to adhere for 4–15 min, rinsed twice with distilled water, and stained for 60–90 s with 5 μl of 1% uranyl acetate (Ted Pella, Inc.). All samples were imaged at an accelerating voltage of 80 kV in a JEOL JSM 1400 Plus (JOEL). Grids from two to four independent experiments were examined.

### Biological replicates and statistical analysis

All cell-based assays were performed in triplicate and in at least three separate experiments. *N* represents technical replicates derived from separate wells of cells treated the same, or individual neurons tracked in longitudinal survival experiments. Statistical analyses were performed in GraphPad Prism v.9 unless otherwise specified.

## RESULTS

### Relative abundance of RAN products is elongation, not initiation, dependent

**Figure 1.**
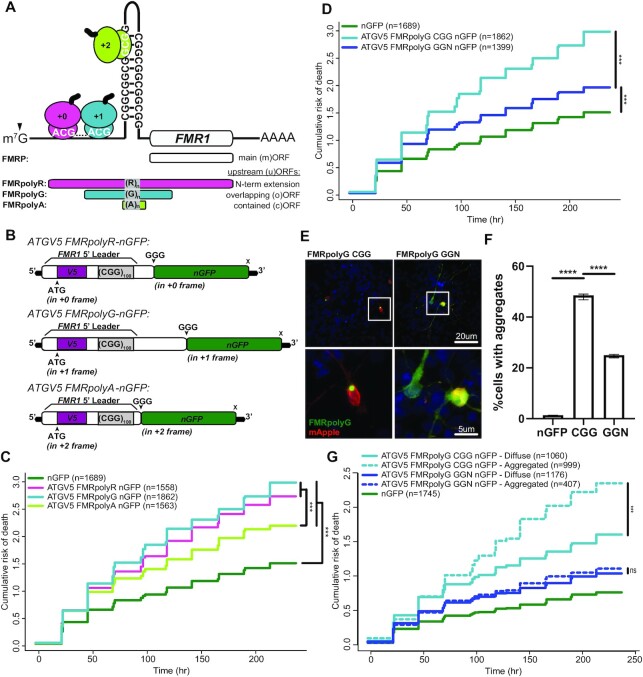
Translation of CGG repeats is neurotoxic in all three reading frames. (**A**) CGG repeats in the 5’UTR of *FMR1* induce ribosome stalling and support RAN translation in different reading frames predominantly at specific near-AUG cognate codons. Initiation at an ACG in the + 0-frame (pink) produces a poly-arginine containing N-terminal extension on FMRP (FMRpolyR). Initiation at ACG or GUG codons in the +1-frame (light blue) produces a poly-glycine (FMRpolyG) peptide that terminates within the first exon of *FMR1*. Initiation within the repeat supports +2 frame (green) production of poly-alanine peptide (FMRpolyA). (**B**) Schematic of reporters with an ATG driving expression through an N-terminal V5 (purple) tag in each reading frame, followed by a C-terminal nGFP (green) tag fused to the 5’UTR of *FMR1* (ATGV5-FMRpolyX-nGFP). (**C**) Survival analysis by longitudinal fluorescence microscopy on rat cortical neurons transfected on (DIV4) with indicated ATGV5-FMRpolyX-nGFP or a control nGFP reporter. ATG-driven translation through the repeat is toxic relative to nGFP (polyR: *P*< 2e–16; polyG: *P*< 2e–16; polyA: *P*< 2e–16), and polyA is significantly less toxic than polyR (*P* = 6.01e–05) or polyG (*P* = 4.63e–09). (**D**) Survival analysis by longitudinal fluorescence microscopy on primary rat cortical neurons expressing ATGV5-FMRpolyG-nGFP through a CGG repeat (light blue) or through an alternating codon (GGN) repeat (navy blue) relative to nGFP (CGG: *P*< 2e–16; GGN *P* = 2.89e–12) (CGG v. GGN: *P* = 6.92e–16). (**E**) Representative confocal images of ATGV5-FMRpolyG-nGFP reporter expression in rat cortical neurons (DIV4). 60X images, Scale bars, 20 μm (inset, 5 μm). (**F**) Percentage of neurons identified in survival analysis with ATGV5-FMRpolyG-nGFP inclusions, as determined by GFP CV measurements (nGFP v. CGG: *P*< 0.0001; CGG v. GGN: *P*< 0.0001) (total n: nGFP = 1689, CGG = 1862, GGN = 1399). (**G**) Survival analysis by longitudinal fluorescence microscopy on primary rat cortical neurons expressing ATGV5-FMRpolyG-nGFP through a CGG repeat (light blue) or through a GGN repeat (navy blue), comparing survival of cells with aggregates (dotted line) or without aggregates (solid line) (CGG: *P* = <2e–16; GGN: *P* = 0.38). (C, D, G) *n*, number of neurons; Cox proportional hazard analysis. (F) Two-sided unpaired Student's *t*-test, graphs are mean ± 95% confidence interval. ns = ‘not significant’, ***P< 0.001, ****P< 0.0001.

RAN translation at CGG repeats can theoretically occur in all three reading frames to produce FMRpolyR (+0), FMRpolyG (+1) and FMRpolyA (+2) (Figure 1A) (22). To evaluate the relative abundance of these RAN events in HEK293Ts, we generated reporters containing the 5’UTR of *FMR1* with a CGG_100_ repeat upstream of a mutated nanoluciferase that lacks an AUG start codon and 3xFLAG tags in each reading frame to allow for quantification of RAN translation by luciferase assay and western blot, respectively (CGG_100_-pX-NL3xFLAG) (Supplemental Figure 1A). As shown previously (22), polyG-frame RAN translation was most abundant, followed by polyA-frame, and polyR-frame translation, at 37.6% and 2.5% relative to polyG, respectively (Supplemental Figure 1B) (22). To determine if different RAN levels across different reading frames was due to initiation efficiency or elongation rates, we introduced an ATG in optimal Kozak sequence context into the 5’UTR in the reading frame corresponding to the downstream luciferase reporter (Supplemental Figure 1A). When potential differences in initiation efficiency were eliminated, relative RAN production in each reading frame was largely unaffected (Supplemental Figure 1C) (22,43). This suggests that translational efficiency differences across CGG repeat reading frames are strongly influenced by differences in the rate of translational elongation through the CGG repeat, consistent with findings in other repeat expansion disorders (43,44).

### AUG-initiated CGG repeat translation is neurotoxic in all three reading frames

To evaluate the relative toxicity of different CGG RAN translation products, we generated reporters containing the 5’UTR of *FMR1* with a CGG_100_ and an ATG codon in a strong Kozak sequence context upstream of the repeat to drive translation in each reading frame. These were fused to green fluorescent protein with its ATG start codon mutated to GGG (nGFP) (Figure 1B). We co-transfected these ATGV5-FMRpolyX-nGFP reporters with mApple into primary rat cortical neurons and imaged them by longitudinal microscopy for ten days to determine the cumulative risk of death as a measure of neuronal survival (38,45–48).

Despite different abundances, AUG-driven FMRpolyR, FMRpolyG and FMRpolyA expressing constructs were all significantly toxic in neurons, with higher cumulative risk of death compared to an nGFP control (Figure 1C). FMRpolyA was less toxic than FMRpolyR or FMRpolyG (Figure 1C). Confocal images of transfected neurons showed that each RAN product had a distinct localization pattern that is largely consistent with prior studies in non-neuronal cells (Supplemental Figure 1D) (21,26,30,49). FMRpolyR localized in the soma, nucleus, and nucleolus. FMRpolyG formed intranuclear inclusions. FMRpolyA exhibited a diffuse distribution throughout the neuron. These data suggest that FMRpolyR and FMRpolyA have the potential to contribute to CGG repeat triggered neurotoxicity.

### FMRpolyG generated from non-repetitive alternating codons is less toxic

To try and evaluate the impact of FMRpolyR or FMRpolyG in isolation, we used the degeneracy of the codon table to generate a poly-arginine or poly-glycine sequence that lacked the CGG repeat and does not support RAN translation in other reading frames. The ATGV5-FMRpolyR-CGN-nGFP repeat ‘CGN’ represents a mixture of all 6 potential arginine codons (AGA, AGG, CGC, CGG, CGA, CGT), while the ATGV5-FMRpolyG-GGN-nGFP (where N = any nucleotide) construct used a mixture of all 4 potential glycine codons (GGC, GGG, GGA, GGT). These constructs were designed to limit RNA secondary structure formation.

Expression of FMRpolyR from an alternative codon (CGN) was slightly, yet significantly, more toxic than FMRpolyR produced from a pure CGG repeat (Supplemental Figure 2A), despite no significant change in expression of FMRpolyR as determined by GFP fluorescence (Supplemental Figure 2B) or mRNA levels (Supplemental Figure 2C).

ATG-driven FMRpolyG expression from an alternating codon sequence (GGN) was significantly less toxic than ATG-driven FMRpolyG constructs containing a pure CGG repeat, though it still increased the cumulative risk of death in neurons relative to GFP (Figure 1D). This decrease in toxicity occurred despite FMRpolyG-GGN leading to more FMRpolyG production than FMRpolyG-CGG (Supplemental Figure 2D). FMRpolyG-GGN did generate lower levels of mRNA expression (Supplemental Figure 2E), indicating that FMRpolyG translation through an alternating-codon repeat is more efficient than through a pure CGG repeat (28).

CGG repeat RNA can interact with RNA binding proteins and impair their native functions (17,18,20,50). To assess whether the reduced toxicity of FMRpolyG produced via GGN translation might be explained by the lack of CGG repeat RNA, we mutated critical near-cognate codons in the glycine reading frame and introduced a stop codon just upstream of the CGG repeat to block most FMRpolyG translation (Supplemental Figure 2F), without affecting RNA levels (21,51). Neurons transfected with this stop@-12 ‘RNA only’ FMRpolyG showed significantly lower risk of death compared to neurons expressing the native 5’UTR of *FMR1* with 100 CGG repeats, which supports RAN translation, consistent with prior studies (21,27). The ‘CGG RNA only’ construct was slightly more toxic than nGFP alone (Supplemental Figure 2F) (21,27). The apparently mild toxicity of CGG RNA alone suggests that the large reduction in toxicity of FMRpolyG when expressed from a GGN repeat is unlikely to be explained by a lack of CGG repeat RNA elicited toxicity.

To understand how differences in the repeat sequence might influence FMRpolyG toxicity, we used confocal microscopy to determine the distribution of these proteins in transfected neurons. Consistent with previously published results, FMRpolyG-CGG formed GFP positive intranuclear inclusions; however, FMRpolyG produced from GGN translation was less likely to form inclusions and instead exhibited a more diffuse localization pattern throughout the neuron (Figure 1E) (21,28,29).

Formation of inclusions was previously shown to be protective for neurons expressing a polyglutamine fragment of *HTT* (39). To evaluate whether FMRpolyG aggregates were associated with toxicity or protection, and to quantify the propensity of aggregation in CGG versus GGN FMRpolyG constructs, we used the coefficient of variation (CV), a measure of the spatial variability of fluorescence across a cell that is directly proportional to aggregation (Supplemental Figure 2G) (41,42). For neurons expressing FMRpolyG constructs, a CV threshold of 1.0 correctly identifies cells with aggregates and those without aggregates (‘diffuse’) with 95.9% sensitivity and 93.8% specificity (Supplemental Figure 2G). As observed qualitatively by confocal imaging (Figure 1E), FMRpolyG-CGG was more likely to aggregate (48.5% of cells containing aggregates) than FMRpolyG-GGN (25.0%) (Figure 1F), while erroneous detection of aggregates in nGFP-expressing cells was low (1.4%). FMRpolyG-CGG expressing neurons exhibiting aggregates showed enhanced rates of death compared to those with only diffuse FMRpolyG-CGG. In contrast, neurons expressing FMRpolyG-GGN did not show differences in neuronal survival between those exhibiting aggregates and those that did not (Figure 1G). This suggests that the reduced toxicity observed in the FMRpolyG-GGN construct reflects both a reduction in aggregate formation and a qualitative difference in the toxicity of aggregates derived from this degenerate codon sequence.

### PolyR-frame RAN initiation contributes to FMRpolyG production

**Figure 2.**
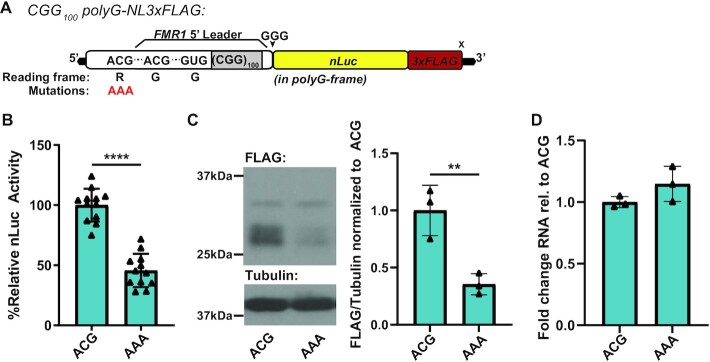
polyR RAN initiation contributes to polyG translation. **(A**) Schematic of CGG_100_-polyG-NL3xFLAG reporter, contains full 5’UTR of *FMR1* (white) with CGG_100_ repeat (gray) fused to nanoluciferase (yellow) and 3xFLAG (red) tags in polyG (+1) reading frame. polyR-frame ACG was mutated to AAA (red) to block polyR translation. (**B**) CGG_100_-polyG-NL3xFLAG reporter nanoluciferase activity with native polyR-frame ACG or AAA mutation in HEK293T cells (ACG versus AAA: *P*< 0.0001, *n* = 12) (**C**) CGG_100_-polyG-NL3xFLAG reporter 3xFLAG representative immunoblot with native polyR-frame ACG or AAA mutation in HEK293T cells, quantified at right as FLAG signal from all bands normalized to Tubulin (ACG versus AAA: *P* = 0.0094, *n* = 3). (**D**) CGG_100_-polyG-NL3xFLAG reporter mRNA expression in HEK293T cells by RT-qPCR (*P* = 0.1637, *n* = 3). b,c,d. two-sided unpaired student's t-test. Graphs are mean ± stdev. **P< 0.01, ****P< 0.0001.

Reduced toxicity and aggregation of GGN-translated FMRpolyG could result from the inability of this construct to support RAN translation in other reading frames. To investigate the possibility that RAN translation in different reading frames influences FMRpolyG-associated toxicity, we used our previously described RAN translation-specific reporters with nanoluciferase and 3xFLAG tags (CGG_100_-polyG-NL3xFLAG) (Figure 2A) (22,33). The polyG product translated from these reporters has an expected molecular weight of 31.47 kDa protein (Supplemental Table 1), but exhibits a multi-band pattern with some bands being larger than predicted, as previously described (22,33).

To study the interaction among reading frame initiation events, we mutated the previously described polyR-frame initiation site (ACG) to AAA to preclude initiation from this codon (22). As this codon is 5’ to the major initiation sites in the polyG-frame, we predicted that this mutation would enhance translation in the polyG reading frame. This construct instead exhibited significantly reduced polyG-frame translation by luciferase assay (Figure 2B), and western blot (Figure 2C). The ACG to AAA mutation did not interfere with the surrounding sequence context of any defined FMRpolyG initiation codons and did not change the predicted RNA structure (Supplemental Figure 3A). Moreover, reporter RNA levels were unaffected by this mutation as measured by RT-qPCR (Figure 2D). We also observed reduced polyG-frame translation with the polyR-frame initiation site mutated at shorter repeats (CGG_25_, Supplemental Figure 3B) and when *in vitro* transcribed RNA reporters were transfected into HEK293T cells to bypass any potential transcriptional or RNA transport elicited effects (Supplemental Figure 3C). These results suggested that polyR-frame RAN initiation significantly and directly contributes to translation in the polyG-frame.

### CGG repeats support robust translational frameshifting *in vitro*

**Figure 3.**
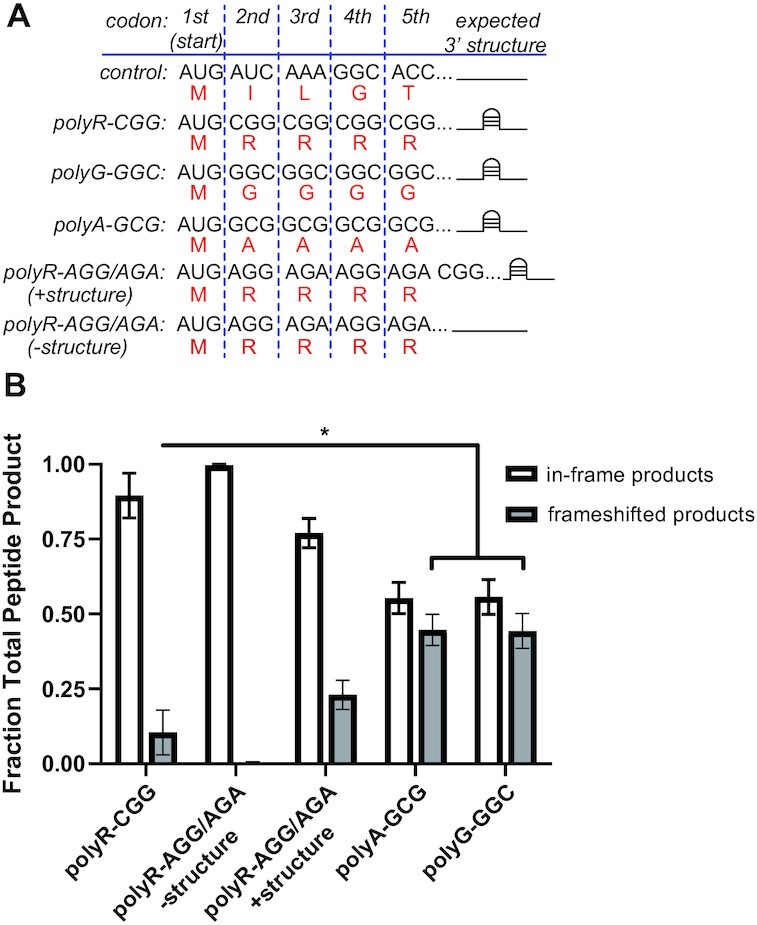
*In vitro* translation of CGG repeats support frameshifting. (**A**) *In vitro* transcribed RNAs (black) used for reconstituted in vitro translation assay (expected amino acid sequence in red). (**B**) Quantification of in-frame versus frameshifted products *in vitro* translation assay (*n* = 3) (polyR-CGG versus polyG-GGC: *P* = 0.0482; polyR-CGG versus polyA-GCG: *P* = 0.0423; polyA-GCG versus polyG-GGC: *P* = 0.9680). (B) Two-sided unpaired Student's *t*-test. Graphs are mean ± sem. *P< 0.05.

One potential explanation for the impact of CGG repeats on differential toxicity and on the loss of polyG signal when initiation in the polyR frame is impaired is that these repeats could support a translational frameshift event. To assess the possibility of frameshifting on CGG repeats, we first utilized a fully reconstituted *E. coli in vitro* translation assay that can resolve short ^35^S-methionine-labeled translation products by electrophoretic thin layer chromatography (eTLC). We evaluated the translation of three short mRNA sequences containing CGG repeats in each reading frame (polyR-CGG, polyG-GGC and polyA-GCG) following an upstream AUG. We also conducted translation assays with two additional polyR sequences generated without CGGs to evaluate the role of amino acid charge, given that arginine is positively charged and thought to interfere with elongation by interacting with the negatively charged ribosome exit tunnel (52,53). In addition, as the CGG repeat forms a strong hairpin structure, the two polyR-AGG/AGA constructs were generated such that they either have downstream CGG repeats, expected to form a 3’ hairpin, or an unstructured sequence. We compared the translation of these five sequences to a control MIKG-encoding sequence (Figure 3A) that is easily translated to estimate the extent of ribosome frameshifting at CGG repeats. These experiments enabled us to begin deconvoluting the contributions of RNA sequence, amino acid charge, and/or the 3’ secondary structure of the RNA to any observed frameshifting events.

We readily detected positively charged products in polyA-GCG and polyG-GGC samples and uncharged products in polyR-CGG, indicative of widespread frameshifting at CGG repeats in all reading frames (Figure 3B; Supplemental Figure 4). A greater proportion of ribosomes frameshifted on polyG-GGC and polyA-GCG messages (44%) than on analogous polyR-CGG messages (10%) (Figure 3B). Frameshifting on polyR-AGG/AGA sequences in the absence of downstream structure was nearly undetectable (<1%). However, when a downstream CGG repeat structured element was present, frameshifting increased to levels comparable to that observed on the polyR-CGG mRNA (Figure 3B). Together, these data suggest that translation through CGG repeats is highly prone to ribosomal frameshifts. Moreover, these frameshift events are dependent primarily on the CGG repeat sequence itself and are enhanced by downstream RNA secondary structures that might slow ribosomal elongation.

### CGG repeats support an R-to-G +1 translational frameshift in cells

**Figure 4.**
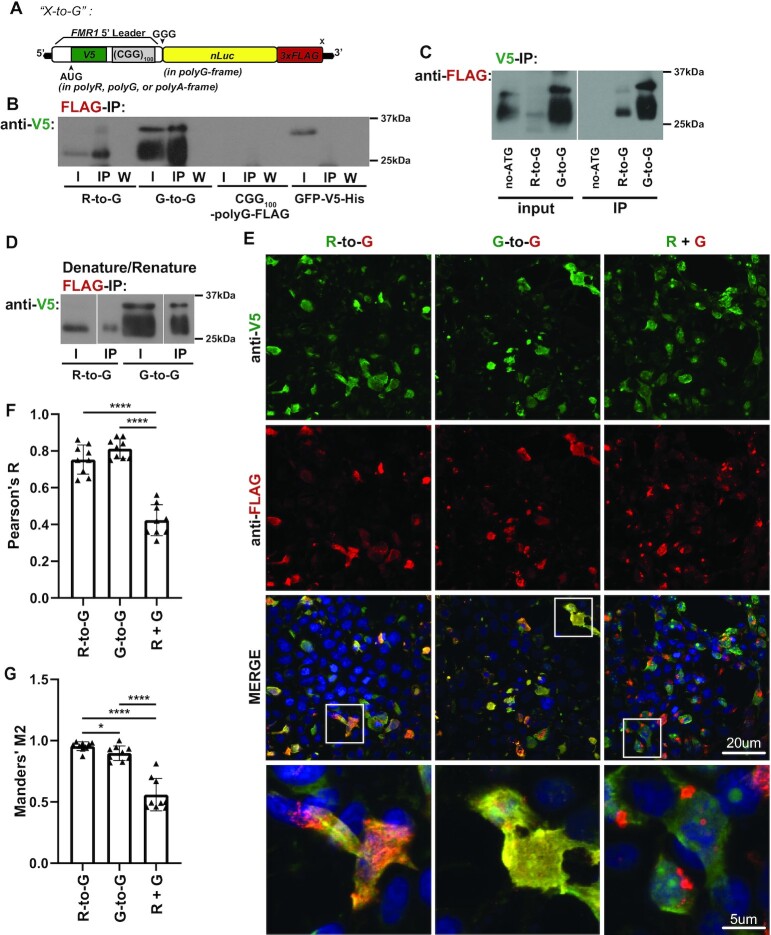
CGG repeats support *in vivo* R-to-G +1 translational frameshift. (**A**) Schematic of N/C-terminal dual tagged reporters with an ATG driving translation in each reading frame through an N-terminal V5 tag (green) contained within the 5’UTR of *FMR1* (white) upstream of a CGG_100_ repeat, fused to C-terminal nanoluciferase and 3xFLAG tags in the polyG reading frame (‘X-to-G’, X = frame of N-terminal tag, G = frame of C-terminal tag in the polyG-frame). (**B**) X-to-G reporters expressed in HEK293T cells were immunoprecipitated for C-terminal FLAG tag and immunoblotted for N-terminal V5 tag. Blot representative of experiment performed in triplicate. (I = input, IP = immunoprecipitated, W = wash.) (**C**) X-to-G plasmid reporters expressed in HEK293T cells were immunoprecipitated for N-terminal V5 tag and immunoblotted for C-terminal FLAG tag. (**D**) Denature-renature IP of X-to-G reporters expressed in HEK293T cells. Lysates were treated with SDS to denature any peptide-peptide interactions before dilution and IP of C-terminal FLAG tag, immunoblotted for N-terminal V5 tag. (**E**) R-to-G and G-to-G dual-tagged reporters were expressed in HEK293T cells, along with co-expression of ATGV5-polyR and ATG-polyG-NL3xFLAG, and stained for V5 (green), FLAG (red), and DAPI (blue) for co-localization analysis. Representative images. 60× images, Scale bars, 20 μm (inset, 5 μm). (**F**) Pearson's R coefficient (i.e. the linear correlation between the signal changes in two channels across an image) of R-to-G, G-to-G, and co-expressed polyR and polyG (rel. to ‘R + G’, R-to-G: *P*< 0.0001; G-to-G: *P*< 0.0001) (*n* = 9 images/group). (**G**) Manders’ M2 coefficients (i.e. the ratio of FLAG-positive pixels that have V5 signal) of R-to-G, G-to-G, and co-expressed polyR and polyG (R-to-R versus G-to-G *P* = 0.0252; rel. to ‘R + G’, R-to-G: *P*< 0.0001, G-to-G: *P*< 0.0001) (*n* = 9 images/group). (B, C) Two-sided unpaired Student's *t*-test. Graphs are mean ± stdev. *P< 0.05, ****P< 0.0001.

As our *in vitro* evidence suggested that frameshifting can occur at CGG repeats, we sought to evaluate frameshifting *in vivo*. We developed a series of N/C-terminal dual tagged reporters with an ATG driving translation in each reading frame through an N-terminal V5 tag contained within the 5’UTR of *FMR1* upstream of a CGG_100_ repeat, fused to C-terminal nanoluciferase and 3xFLAG tags in each reading frame (‘X-to-Y’, X = frame of N-terminal tag, Y = frame of C-terminal tag) (Figure 4A, Supplemental Figure 5A).

To evaluate the possibility of frameshifting from these dual-tagged X-to-Y reporters, we immunoblotted for either the N-terminal V5 or the C-terminal FLAG tags to identify bands positive for both, despite the tags being in different reading frames. As expected, all reporters with in-frame N- and C-terminal tags had dual-positive bands of the same product size (Supplemental Figure 5B). Additionally, when translation initiation was driven in the polyR-frame, with a C-terminal tag in the polyG-frame, a 25 kDa band showed signal for both V5 and FLAG. We reasoned that this dual-positive band could represent an R-to-G +1 frameshifted product consistent with our observation that polyR-frame initiation contributes to polyG-frame translation. However, unlike our *in vitro* studies, we did not observe robust frameshifting in other reading frames, suggesting that the +1 frameshift may be favored in mammalian cellular contexts (Supplemental Figure 5B).

To validate this R-to-G frameshift, we immunoprecipitated using antibodies that react with the C-terminal FLAG tag. We detected the presence of a V5-positive band by immunoblot (Figure 4B) and the presence of a V5-peptide by Mass Spectrometry (MS) (Supplemental Figure 5C). We also pulled down using V5 antibodies and detected a FLAG-positive band by immunoblot (Figure 4C). In both cases, a dual-positive band was detected for the positive control construct in which the N-terminal and C-terminal tags are both in the polyG-frame (G-to-G), while negative control constructs containing only a FLAG (CGG_100_-polyG-FLAG) or only a V5 tag (GFP-V5-His) either did not pull down or did not stain for the absent tag, as expected.

To confirm that these R-to-G immunoprecipitations reflect chimeric frameshifted products and not peptide-peptide interactions of pure polyR and polyG products, we denatured the protein lysate in sodium dodecyl sulfate (SDS) to disrupt any non-covalent interactions before pull-down using antibodies against the C-terminal FLAG tag (21,54). Immunoblotting for the N-terminal tag still showed a single dual-positive band in the R-to-G frameshift reporter (Figure 4D), suggesting that this band represents a single chimeric frameshifted product and not a pure co-immunoprecipitated polyG.

As a second test for ribosomal frameshifting, we expressed our R-to-G frameshift reporter, a G-to-G dual-tagged in-frame reporter, or a mixture of ATG-driven polyG and polyR reporters in HEK293T cells and stained for V5 (green) and FLAG (red) to assess for co-localization (Figure 4E). We reasoned that frameshift proteins would be highly overlapped in their signals while the separately expressed products would show partial or no colocalization. Consistent with this prediction, V5 and FLAG signals from the R-to-G and G-to-G reporter were highly correlated by Pearson's coefficient (Figure 4F). The overlap in their signals (Manders’ M2) was significantly greater than when polyR and polyG were expressed from separate reporters (Figure 4G). Together, these data provide orthogonal evidence of a R-to-G translational frameshift in the 5’UTR of *FMR1*.

### The R-to-G translational frameshift primarily produces a polyG peptide

**Figure 5.**
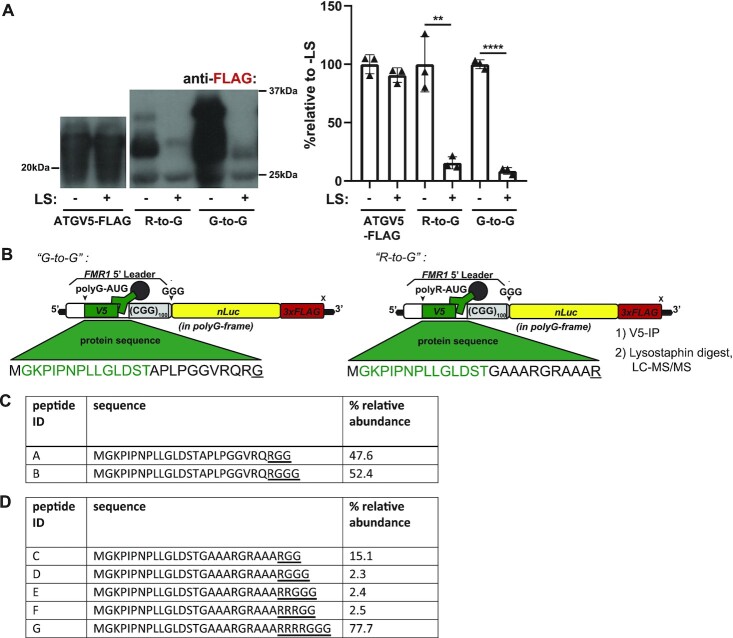
R-to-G translational frameshift within CGG repeat produces polyglycine containing peptide. (**A**) R-to-G, G-to-G and CGG-lacking (‘ATGV5-FLAG’) dual-tagged reporters were expressed in HEK293T cells, lysates treated with lysostaphin (LS). Representative FLAG immunoblots, all bands quantified together at right (*n* = 3; R-to-G: *P* = 0.0038, G-to-G: *P*< 0.0001). (**B**) Schematic of LC–MS/MS experiment, in which G-to-G and R-to-G reporter was expressed in HEK293T cells, lysates pulled down for N-terminal V5 tag, and treated with LS to isolate N-terminal frameshifted peptide fragments. Peptide sequence resulting from translation into CGG repeat is underlined. (**C**) Table showing fragments identified by LC-MS/MS and their relative abundance in G-to-G in-frame dual-tagged samples. Peptide sequence resulting from translation into CGG repeat is underlined. (**D**) Table showing frameshifted fragments identified by LC-MS/MS and their relative abundance in R-to-G samples. Peptide sequence resulting from translation into CGG repeat is underlined. a. two-sided unpaired Student's *t*-test. Graphs are mean ± stdev. **P< 0.01, ****P< 0.0001.

To determine if the R-to-G frameshifted peptide contains a poly-glycine stretch, we expressed X-to-G constructs in HEK293T cells and incubated lysates with lysostaphin, an endopeptidase that selectively cleaves at pentaglycine stretches (GG/GGG). We then immunoprecipitated with the N-terminal V5 tag and immunoblotted samples for the C-terminal FLAG tag. A reporter lacking a CGG repeat (ATGV5-FLAG) did not degrade with lysostaphin treatment. In contrast, both the R-to-G and G-to-G reporters almost completely degraded (Figure 5A). This suggests that the R-to-G frameshift event occurs within the CGG repeat to generate a primarily polyG, rather than primarily a polyR, containing protein.

### The R-to-G frameshift occurs within the CGG repeat

To identify the exact site of frameshifting, we immunoprecipitated lysates from HEK293T cells expressing the R-to-G frameshift reporter using a V5 antibody and treated the immunoprecipitated fraction with lysostaphin to isolate N-terminal protein fragments that may be detectable by MS (Figure 5B). Using this method, we detected the expected N-terminal fragment in the G-to-G sample (GKPIPNPLLGLDSTAPLPGGVRQRGG/G; 47.6%, 52.4% relative abundance, respectively) (Figure 5C, Supplemental Figure 6A). When using the R-to-G frameshifted reporters, we readily detected evidence of frameshifting into the polyG reading frame (Figure 5D). The most abundant product detected (Supplemental Figure 6B; GKPIPNPLLGLDSTGAAARGRAAARRRRG; 77.7% relative abundance) indicates that a frameshift occurs preferentially after incorporating four arginines, but can occur after 1, 2 or 3 arginines at relative rates of 17.4, 2.4 and 2.5%, respectively (Figure 5D). While this confirms that frameshifts occur after just a few CGG codons *in vivo*, it is worth noting here that we may not be able to detect longer fragments generated from frameshifts further into the repeat sequence using this method.

### Driving polyR-translation initiation enhances FMRpolyG signal and neurotoxicity

**Figure 6.**
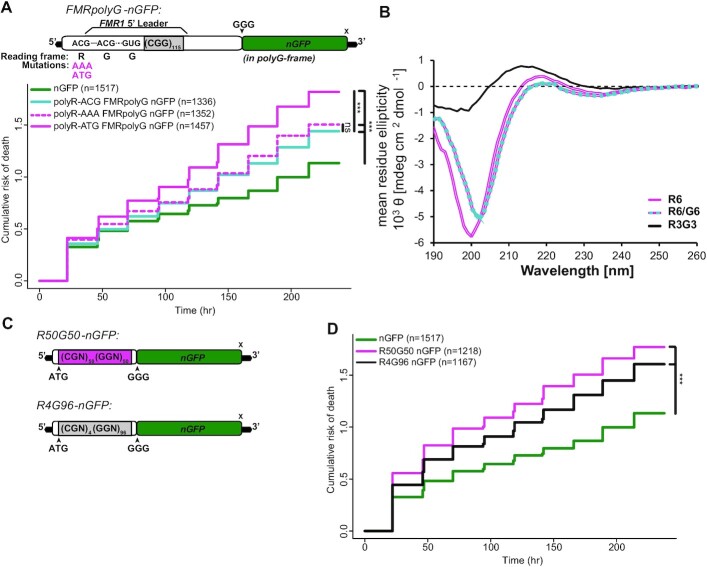
Chimeric peptides exhibit unique biophysical properties and induce neurotoxicity. (**A**) Top: schematic of FMRpolyG-nGFP reporter with CGG_115_, and polyR-frame ACG mutated to AAA to preclude translation initiation and to ATG to promote translation initiation. Bottom: survival analysis by longitudinal fluorescence microscopy on primary rat cortical neurons expressing FMRpolyG with native polyR-ACG (blue), or with ACG mutated to AAA (dotted pink) or ATG (pink) (relative to nGFP (green); polyR-ACG: *P*< 4.82e-06; polyR-AAA: *P* = 7.73e−09, polyR-ATG: *P*< 2e−16) (polyR-ACG v polyR-AAA: *P* = 0.235; polyR-ACG versus polyR-ATG: *P* = 9.42e−08). (**B**) CD measurements show that the spectra of R6 (0.3mg/mL, pink) and R6/G6 mixtures (0.3 mg/mL of each peptide, pink/blue dashed line) differ, suggesting that the secondary structure of R6 is altered in the presence of G6. The spectra of chimeric R3G3 (0.3 mg/ml, black) is also distinct than R6 and R6/G6 mixture. Spectra of G6 is not shown because glycine is achiral and does not produce CD signal. (**C**) Schematic of polymer expression vectors with optimized alternative codon sequences to express 50 arginines followed immediately by 50 glycines (R50G50), and four arginines followed by 96 glycines (R4G96) upstream of GFP. (**D**) Survival analysis by longitudinal fluorescence microscopy on primary rat cortical neurons expressing polymers R50G50 (pink) or R4G94 (black) (relative to nGFP (green): R50G50: *P*< 2e−16, R4G96: *P* = 4.36e−15). (A, D) *n*, number of neurons; Cox proportional hazard analysis. n.s. = not significant, ***P< 0.001.

To evaluate the potential contribution of R-to-G frameshifting on FMRpolyG neurotoxicty, we generated FMRpolyG-nGFP reporters with a CGG_115_ and introduced mutations to the polyR-ACG. The polyR-AAA mutation should preclude initiation and block frameshifting. As expected, polyR-AAA reduced FMRpolyG-nGFP fluorescence in neurons (Supplemental Figure 7A). However, this mutation did not significantly alter neuronal survival compared to the native polyR-ACG construct (Figure 6A). Placing an ATG codon in the ACG site to drive initiation in polyR reading frame resulted in enhanced FMRpolyG-nGFP signal in neurons (Supplemental Figure 7A). This AUG codon significantly increased the risk of neuronal death compared to the native polyR-ACG construct (Figure 6A). Together, these data suggest that enhancing the R-to-G frameshifting event increases CGG repeat toxicity.

### Chimeric R3G3 peptide exhibit distinct biophysical properties and induce greater neurotoxicity than pure polyR or polyG peptides

Our data suggests that a subset of polyG-containing RAN products have a polyR string at the N-terminus of the repetitive region. To evaluate if these chimeric polymers have distinct biophysical properties, we generated simple 6mer peptides; a pure polyR (R6), a pure polyG (G6) and a chimeric (R3G3) peptide. Circular dichroism (CD) is a technique in which a suspension is exposed to circularly polarized light. The resulting absorption spectra informs on the secondary structure of a given peptide. Of note, glycine is achiral, so is not detectable by CD. The R6 peptide fit a random-coiled structure (Figure 6B). CD spectra of an R6/G6 mixture differed from that of pure R6, indicating that the secondary structure of R6 is altered in the presence of G6 (Figure 6B). However, the CD spectra of the chimeric R3G3 was quite distinct from either the R6 and R6/G6 mixtures (Figure 6B), with loss of the coiled structure elicited by the R6 peptide. CD spectra for all three samples did not change when peptides were incubated at 37°C to induce aggregation (Supplemental Figure 7B).

Consistent with the differential CD spectra, we also observed differences among these peptide mixtures in their aggregation and secondary structure by transmission electron microscopy (TEM). The chimeric R3G3 peptide formed large sheet-like species that were absent in R6, and very rare in R6/G6 mixtures. R3G3 also formed spherical aggregates that were larger than those observed in G6 samples (Supplemental Figure 7C).

To evaluate whether there might be differential toxicity between pure R or G peptides and chimeric peptides, we added peptides in solution to primary rat cortical neurons transfected with mApple and tracked their survival by longitudinal microscopy. G6 alone showed no toxicity relative to buffer (Supplemental Figure 7D). Pure R6 was slightly toxic, but less so than a R6/G6 mixture (Supplemental Figure 7D). The chimeric R3G3 peptide was the most toxic relative to buffer (Supplemental Figure 7D) and was comparable to that of our positive control, addition of aggregated alpha-synuclein fibrils (Supplemental Figure 7D) (55). Together, these data suggest that chimeric peptides are prone to aggregation, form structures distinct from those formed by pure peptides, and that they exhibit differential toxicity in neurons.

### Chimeric polymers produced via codon-optimized expression vectors are neurotoxic

To evaluate the potential toxicity of chimeric R/G polymers that are more representative of products observed *in vivo*, we generated codon-optimized constructs with an ATG to drive expression of two chimeras. The first (R50G50) chimera has 50 arginines followed immediately by 50 glycines. The second chimera mirrors the major product identified by MS (R4G96) with four arginines followed immediately by 96 glycines, all placed upstream of GFP (Figure 6C). Both chimeras were toxic when expressed in rodent neurons compared to nGFP (Figure 6D), suggesting that R/G polymers can confer significant neurotoxicity.

## DISCUSSION

Over the past decade, RAN translation has emerged as an important pathogenic contributor in repeat-associated neurodegenerative disorders (20,21,23,56–58). As RAN translation has the potential to generate toxic proteins from all three reading frames in both the sense and antisense directions (20), how each RAN product impacts neurotoxicity in isolation or through toxic combinatorial effects with repeat RNA and other RAN products could have important implications for understanding the pathophysiology of these disorders. Our work here demonstrates that frameshifting at CGG repeats adds an additional layer of complexity to RAN translation-associated toxicity. Chimeric polypeptides exhibit distinct biophysical properties and toxicity compared to the pure homopolymeric proteins. Moreover, toxicity of chimeric products comparable to that of pure RAN products imply that interplay between different reading frames during RAN translation and after RAN product generation may be important contributors to neurodegeneration in FXTAS and related disorders.

The + 1 frameshift we observe here is consistent with prior mechanistic studies. Translational frameshifting in the −1 direction typically occurs at programmed heptameric ‘slippery sequences’ (i.e. X XXY YYZ, where XXX and YYY are triplets of the same bases and Z is any base) with surrounding stimulatory elements (59,60). In contrast, +1 frameshifting typically depends on elements that increase ribosome pause time, such as secondary structure, amino acid charge, and/or amino-acyl-tRNA availability (61–65). *In vitro*, frameshifted products are readily observed when CGG repeats are translated in any reading frame, but not at an unstructured polyR-AGG/AGA sequence, suggesting that the CGG repeating sequence itself is the frameshift stimulatory element. While amino acid charge alone does not drive frameshifting in the *in vitro* system, the presence of a secondary RNA structure 3’ to the translated sequence significantly boosts frameshifting, as expected.

*In vivo*, we were only able to readily detect frameshifting from the polyR to polyG reading frame. This suggests that factors found in intact cells may favor specific frameshifts at CGG repeats. Perhaps *in vivo* translation is more susceptible to ribosome stalling via the consecutive incorporation of positively charged amino acids (66–68). Moreover, the lower abundance of tRNAs decoding the arginine CGG codon relative to the glycine GGC-decoding tRNA, especially in the context of consecutive arginine translation, may further encourage ribosome pausing and a selective switch into the glycine frame *in vivo* (62,63,69). Of note, our selective detection of an R-to-G frameshift does not rule out other frameshift events, which we suspect may be detectable by different techniques.

Examples of frameshifting in eukaryotes are limited, and recent studies suggest caution when using reporters to characterize such events (70,71). Belew et al. (2014) reported efficient programmed −1 ribosomal frameshifting at a slippery sequence in the CCR5 gene. In this study, authors measured frameshifting as a renilla-to-firefly luciferase ratio from dual-tagged luciferase reporters with an N-terminal renilla luciferase and a C-terminal firefly luciferase in an alternate (−1) reading frame (70). However, multiple failures to replicate these findings in other groups demonstrates that signal from dual-tagged reporters can be easily misinterpreted when expressed as normalized ratios, and without effective controls (71). Though our study uses similar dual-tagged reporters to detect frameshifting, we relied primarily on pull-down assays, ensuring that the N- and C-terminal signal we observe is from a single protein of expected size. Furthermore, LC-MS/MS experiments with our dual-tagged reporters confirmed sequence identity of our in-frame G-to-G control and confirmed the presence of the R-to-G frameshifted peptide.

Prior to the discovery of RAN translation, multiple studies suggested that frameshifting can occur at CAG repeat expansions in polyglutamine disorders, Spinocerebellar ataxia type 3 and Huntington's disease (69,72–74). These studies developed antibodies against the predicted C-terminus of peptides in alternate reading frames to detect poly-alanine and poly-serine products in patient cells and tissues (69,72–74). These frameshift events contributed to repeat pathogenesis in cell-based systems (69), but subsequent work demonstrated that these poly-alanine and poly-serine products could alternatively result from RAN translation, not frameshifting (57,58). However, our data clearly demonstrates that the two processes are not mutually exclusive, as we observe a frameshift event confirmed by MS peptide sequence identification and provide evidence that frameshifting contributes to some RAN product generation.

Our findings call to mind recent work on translation of G_4_C_2_ or TG_3_C_2_ repeats from expansions of that cause C9orf72-mediated Amyotrophic lateral sclerosis and frontotemporal dementia (C9 ALS/FTD) and Spinocerebellar ataxia type 36 (SCA36), respectively (75). RAN translation at both of these repeats produces a glycine-proline polypeptide (polyGP), but while polyGP remains soluble and diffuse in SCA36 cells, it aggregates in C9ALS/FTD (75). In this study, polyGP expressed alone, or when co-expressed with polyGA, remains diffuse in cells, but expression of a GA50-GP50 chimera aggregates (75). Our data exhibit a similar pattern, where the chimeric arginine-glycine peptides have differential toxicity, structural properties, and aggregability in cells compared to poly-glycine or arginine alone.

**Figure 7.**
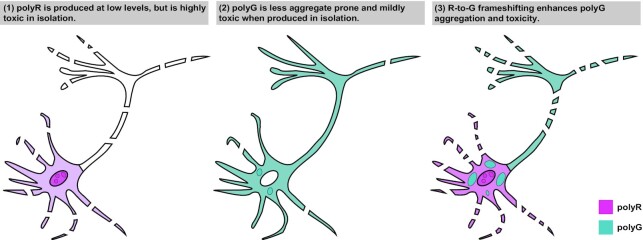
Proposed model: Frameshifted peptides induce FMRpolyG aggregation and toxicity. (**1**) FMRpolyR is produced at low levels in reporter assays and is not readily detectable in patient tissue. When translation in the polyR frame is induced via AUG initiated translation, it is highly toxic to neurons. (**2**) FMRpolyG is the most abundant RAN peptide and accumulates within inclusions in patients. However, when it is translated from an alternate codon construct lacking the CGG repeat that does not support RAN translation or translational frameshifting, it is less prone to aggregation and is less neurotoxic. (**3**) R-to-G frameshifting generates chimeric peptides that have different properties than the pure FMRpolyG protein. We propose that these chimeric peptides serve as a nidus for aggregate formation and FMRpolyG toxicity in FXTAS.

The role of this R-to-G translational frameshift event in FXTAS pathogenesis is unclear. Introducing products that mimic the expected R/G chimera induce toxicity in neurons comparable or greater than the introduction of pure polymers. However, mutations that should block or reduce the R-to-G frameshift (Figure 6A, polyR-AAA) do not significantly reduce FMRpolyG toxicity, nor reduce the inherent toxicity of FMRpolyR (Supplemental Figure 2A). Additionally, introducing a stop codon in the polyG-frame (Supplemental Figure 2F, ‘stop@-12’) significantly reduced FMRpolyG toxicity, though this mutation should not reduce the R-to-G frameshift. Together, these data suggest that the frameshift is a relatively low frequency event, with mild toxicity in isolation, but may result in an increase in total cellular arginine production, that when produced in conjunction with FMRpolyG, enhances toxicity, possibly through aggregate formation (Figure 7). Through mutation of the polyR initiation site, we can estimate the abundance of the R-to-G frameshift to be anywhere between 54% in HEK293T cells (Figure 2B) to as little as 5% in rodent neurons (Supplemental Figure 7A), but further studies will be required to estimate the abundance of the R-to-G frameshift in patient cells and tissues, and their contribution to FXTAS pathology.

Therapeutic efforts in repeat expansion disorders have increasingly targeted RAN translation (14,30,33,76–78). Our work suggests that altering RAN translation in one reading frame may have unintended toxic effects in other frames. Frameshifting at repetitive elements also introduces a new level of complexity when assessing the toxic contributions of different RAN reading frames and peptides. To that end, the current study demonstrates the importance of investigating the production and toxicity of chimeric polymers and the interplay of RAN translation events and frameshifting at repeat expansions.

## DATA AVAILABILITY

Raw Mass Spectroscopy results are available on ProteomeXchange. All other data points are included in the main figures or supplementary data. Raw data used for figure generation are available upon request.

## Supplementary Material

gkac626_Supplemental_Files
