# Supplementary material for: CGG repeats trigger translational frameshifts that generate aggregation-prone chimeric proteins
Source: Nucleic Acids Res. 2022 Jul 29;50(15):8674–89. doi: 10.1093/nar/gkac626 (PMC9410890; doi:10.1093/nar/gkac626)
Supplement: gkac626_Supplemental_Files [file gkac626_supplemental_files.zip › Supplemental Tables.pdf]

Supplemental Table 1. Relevant plasmid sequences. ATG or near-ATG codons are in bold. Repeat or alternative codons are underlined.

Name	vector	Sequence (upstream of indicated tag)	Expected product size (kDa)
		From ATG to start of nGFP tag	
ATGV5-FMRpolyR-CGG-nGFP	pGW	ATGGGTAAGCCTATCCCTAACCCTCTCCTCGGTCTCGATTC<br>TACGGGCGCCGCTGCCAGGGGGC**GTG**CGGCAGCG$(CGG)_8$<br>AGG$(CGG)_{91}$CTGGGCCTCGAGCGCCCGCAGCCCACCTCTC<br>GGGGGCGGGCTCCCGGCGCTAGCAGGGCTGAAGAGAAGA<br>AACCGGGGGATATCCTAGGA	47.95
ATGV5-FMRpolyR-CGN-nGFP	pGW	ATGGGTAAGCCTATCCCTAACCCTCTCCTCGGTCTCGATTC<br>TACGGCGGGCGGCGGGCCG**ACG**GCGAGCGCGGGCGGCG<br>GCGGTG**ACG**GAGGCGCCGCTGCCAGGGGGC**GTG**CGGCA<br>GCGCGCCGTAGACGTCGCAGGCGACGTCGGAGGAGACGA<br>CGCCGTCGGAGACGACGTCGAAGGCGTCGTCGTCGACGG<br>CGCAGGCGTAGAAGACGGCGTCGGCGACGTCGACGGCGG<br>CGTCGACGAAGGCGTCGTCGGCGACGGAGAAGAAGACGA<br>CGGCGTCGGCGCAGGCGTCGACGGCGAAGGAGAAGGCGA<br>CGACGTCGACGGCGCAGGAGGCGTCGTCGGCGACGAAGA<br>AGGCGGCGCCGTCGGCGAAGAAGGCGTCGGCGAAGGAGA<br>CGTCGTCGGCGACGACGTCGTCGACGACGGCGTCGAAGG<br>CTGGGCCTCGAGCGCCCGCAGCCCACCTCTCGGGGGCGG<br>GCTCCCGGCGCTAGCAGGGCTGAAGAGAAGAACCTAGGA	49.09
ATGV5-FMRpolyG-CGG-nGFP	pGW	ATGGGTAAGCCTATCCCTAACCCTCTCCTCGGTCTCGATTC<br>TACGGCGCCGCTGCCAGGGGGC**GTG**CGGCAGCG$(CGG)_8$A<br>GG$(CGG)_{91}$CTGGGCCTCGAGCGCCCGCAGCCCACCTCTCG<br>GGGGCGGGCTCCCGGCACTAGCAGGGCTGAAGAGAAGAT<br>GGAGGAGCTGGTGGTGGAAGTGCGGGGCTCCAATGGCGC<br>TTTCTACAAGGCATTTGAAAGCGGCCCGTTTAAACGGCCGC<br>CCGGGGGATATCCTAGGA	41.16
ATGV5-FMRpolyG-GGN-nGFP	pGW	ATGGGTAAGCCTATCCCTAACCCTCTCCTCGGTCTCGATTC<br>TACGGCGCCGCTGCCAGGGGGC**GTG**CGGCAGCGCGGTGG	41.33

		CGGAGGAGGTGGGGGTGGTGGAGGAGGAGGCGGTGGTG<br>GAGGTGGTGGGGGAGGAGGCGGAGGAGGGGGTGGTGGT<br>GGCGGCGGTGGAGGAGGAGGCGGTGGTGGTGGAGGAGG<br>CGGAGGAGGAGGGGGTGGAGGAGGAGGTGGCGGTGGTG<br>GTGGAGGAGGCGGAGGAGGAGGTGGCGGTGGTGGAGGTG<br>GCGGTGGAGGGGGCGGTGGTGGTGGAGGTGGGGGAGGT<br>GGTGGAGGAGGTGGTGGCGGTGGAGGTGGAGGCGGTGGA<br>GGAGGAGGTGGCGGAGGCGGTGGTGGAGGAGGAGGTTGG<br>GCCTCGAGCGCCCGCAGCCCACCTCTCGGGGGCGGGCTC<br>CCGGCACTAGCAGGGCTGAAGAGAAGATGGAGGAGCTGGT<br>GGTGGAAGTGCGGGGCTCCAATGGCGCTTTCTACAAGGCA<br>TTTGAAAGCGGCCCGTTTAAACGGCCGCCCGGGGGATATC<br>CTAGGA	
ATGV5-FMRpolyA-nGFP	pGW	ATGGGTAAGCCTATCCCTAACCCTCTCCTCGGTCTCGATTC<br>TACGGGGCGCCGCTGCCAGGGGGC**GTG**CGGCAGCG(CGG)<br>$_8$AGG(CGG)$_{91}$CTGGGCCTCGAGCGCCCGCAGCCCACCTCTC<br>GGGGGCGGGCTCCCGGCGGAATTCACCGGGGGATATCCT<br>AGGA	31.96
		From HindIII site to start of tag	
FMRpolyG$_{100}$-nGFP	pGW	AAGCTTGGTACCGAGCTCGGATCCACTAGTCCAGTGTGGT<br>GGAATTCGTTAACAGATCTGCTCAGCTCCGTTTCGGTTTCA<br>CTTCCGGTGGAGGGCCGCCTCTGAGCGGGCGGCGGGCCG<br>**ACG**GCGAGCGCGGGCGGCGGCGGTG**ACG**GAGGCGCCGC<br>TGCCAGGGGGC**GTG**CGGCAGCG(CGG)$_8$AGG(CGG)$_{91}$CTGG<br>GCCTCGAGCGCCCGCAGCCCACCTCTCGGGGGCGGGCTC<br>CCGGCACTAGCAGGGCTGAAGAGAAGATGGAGGAGCTGGT<br>GGTGGAAGTGCGGGGCTCCAATGGCGCTTTCTACAAGGCA<br>TTTGAAAGCGGCCCGTTTAAACGGCCGCCCGGGGGATATC<br>CTAGGA	39.86
Stop@-12 FMRpolyG$_{100}$-nGFP	pGW	AAGCTTGGTACCGAGCTCGGATCCACTAGTCCAGTGTGGT<br>GGAATTCGTTAACAGATCTGCTCAGCTCCGTTTCGGTTTCA<br>CTTCCGGTGGAGGGCCGCCTCTGAGCGGGCGGCGGGCCG<br>**ACG**GCGAGCGCGGGCGGCGGCGGTG**ACG**GAGGCGCCGC	0.74 (to stop)<br>38.6 (assuming initiation at

		TGCCAGGGGGC**TAG**CGGCAGCG(CGG)$_8$AGG(CGG)$_{91}$CTGGGCCTCGAGCGCCCGCAGCCCACCTCTCGGGGGCGGGCTCCCGGCACTAGCAGGGCTGAAGAGAAGATGGAGGAGCTGGTGGTGGAAGTGCGGGGCTCCAATGGCGCTTTCTACAAGGCATTTGAAAGCGGCCCGTTTAAACGGCCGCCCGGGGGATATCCTAGGA	repeat in polyG-frame)
		From T7 transcription start site to start of C-terminal tag	
polyR-ACG (“no-ATG”) CGG$_{100}$-polyG-NL3xFLAG	pcDNA	TAATACGACTCACTATAGGGAGACCCAAGCTGGCTAGCGTTTAAACTTAAGCTTGGTACCGAGCTCGGATCCACTAGTCCAGTGTGGTGGAATTCGTTAACAGATCTGCTCAGCTCCGTTTCGGTTTCACTTCCGGTGGAGGGCCGCCTCTGAGCGGGCGGCGGGCCG**ACG**GCGAGCGCGGGCGGCGGTG**ACG**GAGGCGCCGCTGCCAGGGGGC**GTG**CGGCAGCG(CGG)$_8$AGG(CGG)$_{91}$CTGGGCCTCGAGGATATCAAGATCTGGCCTCGGCGGCCAAGCTTGGCAATCCGGTACTGTTGGTAAAGCCACCGGG	31.47
polyG-frame ATG CGG$_{100}$-polyG-NL3xFLAG	pcDNA	TAATACGACTCACTATAGGGAGACCCAAGCTGGCTAGCGTTTAAACTTAAGCTTGGTACCGAGCTCGGATCCACTAGTCCAGTGTGGTGGAATTCGTTACACC**ATG**GCGCCGCTGCCAGGGGGC**GTG**CGGCAGC(CGG)$_8$AGG(CGG)$_{91}$CTGGGCCTCGAGGATATCAAGATCTGGCCTCGGCGGCCAAGCTTGGCAATCCGGTACTGTTGGTAAAGCCACCGGG	31.47
polyR-AAA CGG$_{100}$-polyG-NL3xFLAG	pcDNA	TAATACGACTCACTATAGGGAGACCCAAGCTGGCTAGCGTTTAAACTTAAGCTTGGTACCGAGCTCGGATCCACTAGTCCAGTGTGGTGGAATTCGTTAACAGATCTGCTCAGCTCCGTTTCGGTTTCACTTCCGGTGGAGGGCCGCCTCTGAGCGGGCGGCGGGCCG**AAA**GCGAGCGCGGGCGGCGGTG**ACG**GAGGCGCCGCTGCCAGGGGGC**GTG**CGGCAGCG(CGG)$_8$AGG(CGG)$_{91}$CTGGGCCTCGAGGATATCAAGATCTGGCCTCGGCGGCCAAGCTTGGCAATCCGGTACTGTTGGTAAAGCCACCGGG	31.47
“no-ATG” CGG$_{100}$-polyR-NL3xFLAG	pcDNA	TAATACGACTCACTATAGGGAGACCCAAGCTGGCTAGCGTTTAAACTTAAGCTTGGTACCGAGCTCGGATCCACTAGTCCAGTGTGGTGGAATTCGTTAACAGATCTGCTCAGCTCCGTTTCGGTTTCACTTCCGGTGGAGGGCCGCCTCTGAGCGGGCGGC	39.35

		GGGCCG**ACG**GCGAGCGCGGGCGGCGGCGGTG**ACG**GAGGCGCCGCTGCCAGGGGGC**GTG**CGGCAGCG(CGG)$_{8}$AGG(CGG)$_{91}$CTGGGCCTCGAGGGG	
polyR-frame ATG CGG$_{100}$-polyR-NL3xFLAG	pcDNA	TAATACGACTCACTATAGGGAGACCCAAGCTGGCTAGCGTTTAAACTTAAGCTTGGTACCGAGCTCGGATCCACTAGTCCAGTGTGGTGGAATTCGTTACACC**ATG**GGCGCCGCTGCCAGGGGGC**GTG**CGGCAGCG(CGG)$_{8}$AGG(CGG)$_{91}$CTGGGCCTCGAGGGG	39.35
"no-ATG" CGG$_{100}$-polyA-NL3xFLAG	pcDNA	TAATACGACTCACTATAGGTAATTAATTAATCAGTCAGGCGCTCAGCTCCGTTTCGGTTTCACTTCCGGTGGAGGGCCGCCTCTGAGCGGGCGGCGGGCCG**ACG**GCGAGCGCGGGCGGCGGCGGTG**ACG**GAGGCGCCGCTGCCAGGGGGC**GTG**CGGCAGCG(CGG)$_{8}$AGG(CGG)$_{91}$CTGGGCCTCGAGCGCCCGCAGCCCACCTCTCGGGGGCGGCTCCCGGCGCGGCCGCGGG	30.6 (assuming initiation within repeat for peptide with 100A N-terminal segment)
polyA-frame ATG CGG$_{100}$-polyA-NL3xFLAG	pcDNA	TAATACGACTCACTATAGGGAGACCCAAGCTGGCTAGCGTTTAAACTTAAGCTTGGTACCGAGCTCGGATCCACTAGTCCAGTGTGGTGGAATTCGTTACACC**ATG**GGCGCCGCTGCCAGGGGGC**GTG**CGGCAGCG(CGG)$_{8}$AGG(CGG)$_{91}$CTGGGCCTCGAGCGCCCGCAGCCCACCTCTCGGGGGCGGCTCCCGGCGCGGCCGCGGG	30.6
Dual-tagged reporters:			
R-to-R	pcDNA	TAATACGACTCACTATAGGGAGACCCAAGCTGGCTAGCGTTTAAACTTAAGCTTGGTACCGAGCTCGGATCCACTAGTCCAGTGTGGTGGAATTCGTAGT**ATG**GGTAAGCCTATCCCTAACCCTCTCCTCGGTCTCGATTCTACGGCGCCGCTGCCAGGGGGC**GTG**CGGCAGCG(CGG)$_{8}$AGG(CGG)$_{91}$CTGGGCCTCGAGGATATCAG ATCTGGCCTCGGG	40.8
R-to-G	pcDNA	TAATACGACTCACTATAGGGAGACCCAAGCTGGCTAGCGTTTAAACTTAAGCTTGGTACCGAGCTCGGATCCACTAGTCCAGTGTGGTGGAATTCGTAGT**ATG**GGTAAGCCTATCCCTAACCCTCTCCTCGGTCTCGATTCTACGGCGCCGCTGCCAGGGGGC**GTG**CGGCAGCG(CGG)$_{8}$AGG(CGG)$_{91}$CTGGGCCTCGAGGA	V5-frame: 20.59 (to STOP) FLAG-frame: 30.43 (from GTG in polyG-frame),

		TATCAAGATCTGGCCTCGGCGGCCAAGCTTGGCAATCCGGTACTGTTGGTAAAGCCACCGGG	14.05 (from internal NL ATG)
R-to-A	pcDNA	TAATACGACTCACTATAGGGAGACCCAAGCTGGCTAGCGTTTAAACTTAAGCTTGGTACCGAGCTCGGATCCACTAGTCCAGTGTGGTGGAATTCGTAGT**ATG**GGTAAGCCTATCCCTAACCCTCTCCTCGGTCTCGATTCTACGGGCGCCGCTGCCAGGGGGC**GTG**CGGCAGCG(CGG)$_8$AGG(CGG)$_{91}$CTGGGCCATCAAGATCTGGCCTCGGCGGCCAAGCTTGGCAATCCGGTACTGTTGGTAAAGCCACCGGG	V5-frame: 24.41 (to STOP) FLAG-frame: 30.9 (from repeat in polyA-frame), 14.05 (from internal NL ATG)
G-to-R	pcDNA	TAATACGACTCACTATAGGGAGACCCAAGCTGGCTAGCGTTTAAACTTAAGCTTGGTACCGAGCTCGGATCCACTAGTCCAGTGTGGTGGAATTCGTAGT**ATG**GGTAAGCCTATCCCTAACCCTCTCCTCGGTCTCGATTCTACGGCGCCGCTGCCAGGGGGC**GTG**CGGCAGCG(CGG)$_8$AGG(CGG)$_{91}$CTGGGCCTCGAGGATATCAGATCTGGCCTCGGG	V5-frame: 13.54 (to STOP) FLAG-frame: 38.41 (from repeat in polyR-frame), 14.05 (from internal NL ATG)
G-to-G	pcDNA	TAATACGACTCACTATAGGGAGACCCAAGCTGGCTAGCGTTTAAACTTAAGCTTGGTACCGAGCTCGGATCCACTAGTCCAGTGTGGTGGAATTCGTAGT**ATG**GGTAAGCCTATCCCTAACCCTCTCCTCGGTCTCGATTCTACGGCGCCGCTGCCAGGGGGC**GTG**CGGCAGCG(CGG)$_8$AGG(CGG)$_{91}$CTGGGCCTCGAGGATATCAAGATCTGGCCTCGGCGGCCAAGCTTGGCAATCCGGTACTGTTGGTAAAGCCACCGGG	32.45
G-to-A	pcDNA	TAATACGACTCACTATAGGGAGACCCAAGCTGGCTAGCGTTTAAACTTAAGCTTGGTACCGAGCTCGGATCCACTAGTCCAGTGTGGTGGAATTCGTAGT**ATG**GGTAAGCCTATCCCTAACCCTCTCCTCGGTCTCGATTCTACGGCGCCGCTGCCAGGGGGC**GTG**CGGCAGCG(CGG)$_8$AGG(CGG)$_{91}$CTGGGCCATCAAGATCTGGCCTCGGCGGCCAAGCTTGGCAATCCGGTACTGTTGGTAAAGCCACCGGG	V5-frame: 10.59 (to STOP) FLAG-frame: 30.9 (from repeat in polyA-frame), 14.05 (from internal NL ATG)
A-to-R	pcDNA	TAATACGACTCACTATAGGGAGACCCAAGCTGGCTAGCGTTTAAACTTAAGCTTGGTACCGAGCTCGGATCCACTAGTCCAG	V5-frame: 13.51 (to STOP)

		TGTGGTGGAATTCGTAGT**ATG**GGTAAGCCTATCCCTAACCCTCTCCTCGGTCTCGATTCTACGGGGCGCCGCTGCCAGGGGGC**GTG**CGGCAGCG$(CGG)_8$AGG$(CGG)_{91}$CTGGGCCTCGAGGATATCAGATCTGGCCTCGGG	FLAG-frame: 38.41 (from repeat in polyR-frame), 14.05 (from internal NL ATG)
A-to-G	pcDNA	TAATACGACTCACTATAGGGAGACCCAAGCTGGCTAGCGTTTAAACTTAAGCTTGGTACCGAGCTCGGATCCACTAGTCCAGTGTGGTGGAATTCGTAGT**ATG**GGTAAGCCTATCCCTAACCCTCTCCTCGGTCTCGATTCTACGGGGCGCCGCTGCCAGGGGGC**GTG**CGGCAGCG$(CGG)_8$AGG$(CGG)_{91}$CTGGGCCTCGAGGATATCAAGATCTGGCCTCGGCGGCCAAGCTTGGCAATCCGGTACTGTTGGTAAAGCCACCGGG	V5-frame: 16.31 (to STOP) FLAG-frame: 30.43 (from GTG in polyG-frame), 14.05 (from internal NL ATG)
A-to-A	pcDNA	TAATACGACTCACTATAGGGAGACCCAAGCTGGCTAGCGTTTAAACTTAAGCTTGGTACCGAGCTCGGATCCACTAGTCCAGTGTGGTGGAATTCGTAGT**ATG**GGTAAGCCTATCCCTAACCCTCTCCTCGGTCTCGATTCTACGGGGCGCCGCTGCCAGGGGGC**GTG**CGGCAGCG$(CGG)_8$AGG$(CGG)_{91}$CTGGGCCATCAAGATCTGGCCTCGGCGGCCAAGCTTGGCAATCCGGTACTGTTGGTAAAGCCACCGGG	33.41
		From HindIII to start of nGFP tag	
polyR-ACG FMRpolyG nGFP (CGG_{115})	pGW	AAGCTTGGTACCGAGCTCGGATCCACTAGTCCAGTGTGGTGGAATTCGTTAACAGATCTGCTCAGCTCCGTTTCGGTTTCACTTCCGGTGGAGGGCCGCCTCTGAGCGGGCGGCGGGCCG**ACG**GCGAGCGCGGGCGGCGGCGGTG**ACG**GAGGCGCCGCTGCCAGGGGGC**GTG**CGGCAGCG$(CGG)_{115}$CGAAGAGCTGGGCCTCGAGCGCCCGCAGCCCACCTCTCGGGGGCGGGCTCCCGGCGCTAGCAGGGCTGAGGAGAAGAAAGAGGAGCTGGTGGTGGAAGTGCGGGGCTCCAATGGCGCTTTCTACAAGGCATTTGAAAGCGGCCGCACCGGTCGCCAACCCCTAGGA	(from polyG-ACG) 40.60
polyR-AAA FMRpolyG nGFP (CGG_{115})	pGW	AAGCTTGGTACCGAGCTCGGATCCACTAGTCCAGTGTGGTGGAATTCGTTAACAGATCTGCTCAGCTCCGTTTCGGTTTCACTTCCGGTGGAGGGCCGCCTCTGAGCGGGCGGCGGGCCG	(from polyG-ACG) 40.60

		**AAA**GCGAGCGCGGGCGGCGGCGGTG**ACG**GAGGCGCCGC<br>TGCCAGGGGGC**GTG**CGGCAGCG$(CGG)_{115}$CGAAGAGCTGG<br>GCCTCGAGCGCCCGCAGCCCACCTCTCGGGGGCGGGCTC<br>CCGGCGCTAGCAGGGCTGAGGAGAAGAAAGAGGAGCTGG<br>TGGTGGAAGTGCGGGGCTCCAATGGCGCTTTCTACAAGGC<br>ATTTGAAAGCGGCCGCACCGGTCGCCAACCCCTAGGA	
polyR-ATG FMRpolyG nGFP (CGG_{115})	pGW	AAGCTTGGTACCGAGCTCGGATCCACTAGTCCAGTGTGGT<br>GGAATTCGTTAACAGATCTGCTCAGCTCCGTTTCGGTTTCA<br>CTTCCGGTGGAGGGCCGCCTCTGAGCGGCGGCGGGCCG<br>**ATG**GCGAGCGCGGGCGGCGGCGGTG**ACG**GAGGCGCCGC<br>TGCCAGGGGGC**GTG**CGGCAGCG$(CGG)_{115}$CGAAGAGCTGG<br>GCCTCGAGCGCCCGCAGCCCACCTCTCGGGGGCGGGCTC<br>CCGGCGCTAGCAGGGCTGAGGAGAAGAAAGAGGAGCTGG<br>TGGTGGAAGTGCGGGGCTCCAATGGCGCTTTCTACAAGGC<br>ATTTGAAAGCGGCCGCACCGGTCGCCAACCCCTAGGA	(from polyG-ACG)<br>40.60
R50G50-nGFP	pGW	AAGCTTGGTACCGAGCTCGGATCCACTAGTCCAGTGTGGT<br>GGAATTCGTTAACAGATCTGCTCAGCTCCGTTTCGGTTTCA<br>CTTCCGGTGGAGGGCCGCCTCTGAGCGGCGGCGGGCCG<br>**ATG**GCGAGCGCGGGCGGCGGCGGTG**ACG**GAGGCGCCGC<br>TGCCAGGGGGC**GTG**CGGCAGCGCGCCGTAGACGTCGCAG<br>GCGACGTCGGAGGAGACGACGCCGTCGGAGACGACGTCG<br>AAGGCGTCGTCGTCGACGGCGCAGGCGTAGAAGACGGCG<br>TCGGCGACGTCGACGGCGGCGTCGACGAAGGCGTCGTCG<br>GCGACGGAGAAGAAGAGGTGGCGGAGGAGGTGGGGGTGG<br>TGGAGGAGGAGGCGGTGGTGGAGGTGGTGGGGGAGGAG<br>GCGGAGGAGGGGGTGGTGGTGGCGGCGGTGGAGGAGGA<br>GGCGGTGGTGGAGGAGGCGGAGGAGGGGGTGGAGGAGG<br>AGGTGGCGGTGGCTGGGCCTCGAGCGCCCGCAGCCCACC<br>TCTCGGGGGCGGGCTCCCGGCGCTAGCAGGGCTGAAGAG<br>AAG**ATG**GAGGAGCTGGTGGTGGAAGTGCGGGGCTCCA**AT**<br>**G**GCGCTTTCTACAAGGCATTTGAAAGCGGCCGCACCGGTC<br>GCCACCCTAGGA	44.62

R4G96-nGFP	pGW	AAGCTTGGTACCGAGCTCGGATCCACTAGTCCAGTGTGGT<br>GGAATTCGTTAACAGATCTGCTCAGCTCCGTTTCGGTTTCA<br>CTTCCGGTGGAGGGCCGCCTCTGAGCGGGCGGCGGGCCG<br>**ATG**GCGAGCGCGGGCGGCGGCGGTG**ACG**GAGGCGCCGC<br>TGCCAGGGGGC**GTG**CGGCAGCGCGCCGTAGACGTGGTGG<br>CGGAGGAGGTGGGGGTGGTGGAGGAGGAGGCGGTGGTG<br>GAGGTGGTGGGGGAGGAGGCGGAGGAGGGGGTGGTGGT<br>GGCGGCGGTGGAGGAGGAGGCGGTGGTGGAGGAGGCGG<br>AGGAGGGGGTGGAGGAGGAGGTGGCGGTGGTGGAGGAG<br>GCGGAGGAGGTGGCGGTGGTGGAGGTGGCGGTGGAGGG<br>GGCGGTGGTGGAGGTGGGGGAGGTGGTGGAGGAGGTGGT<br>GGCGGTGGAGGTGGAGGCGGTGGAGGAGGAGGTGGCGG<br>AGGCGGTGGAGGAGGCTGGGCCTCGAGCGCCCGCAGCCC<br>ACCTCTCGGGGGCGGGCTCCCGGCGCTAGCAGGGCTGAA<br>GAGAAG**ATG**GAGGAGCTGGTGGTGGAAGTGCGGGGCTCC<br>A**ATG**GCGCTTTCTACAAGGCATTTGAAAGCGGCCGCACCG<br>GTCGCCACCCTAGGA	40.06

Supplemental Table 2. mRNA sequences used for in vitro translation assay. Transcribed RNA sequences are as follows, start and stop codons are bolded and the coding sequences are underlined.

Control	AAUGCACUUAUCCUCGCAAGACACCUAUAGUGAGUCGUAUUA**AUG**AUCAAAGGCACCAGCCUGCUGUGCGCGGUGCCUGAGUCCCACGUAGUCCGGCCCCCGUCUGGUGUGAAGCUUGGAACCCUGUGAACGGU**UAA**UGGCUACAGAAGGGC
polyR-CGG	AAUGCACUUAUCCUCGCAAGACACCUAUAGUGAGUCGUAUUA**AUG**CGGCGGCGGCGGCGGCGGCGGCGGCGGCGGCGGCGGCGGCGGCGGCGGCGGCGGCGGCGGCGGCGGCGGCGGCGGCGGCGGCGG**UAA**UGGCUACAGAAGGGC
polyR-AGG/AGA +structure	AAUGCACUUAUCCUCGCAAGACACCUAUAGUGAGUCGUAUUA**AUG**AGGAGAAGGAGAAGGCGGCGGCGGCGGCGGCGGCGGCGGCGGCGGCGGCGGCGGCGGCGGCGGCGGCGGCGGCGGCGGCGGCGG**UAA**UGGCUACAGAAGGGC
polyR-AGG/AGA -structure	AAUGCACUUAUCCUCGCAAGACACCUAUAGUGAGUCGUAUUA**AUG**AGGAGAAGGAGAAGGAGAAGGAGAAGGAGAAGGAGAAGGAGAAGGAGAAGGAGAAGGAGAAGGAGAAGGAGAAGGAGAAGGAGAAGGAGA**UAA**UGGCUACAGAAGGGC
polyA-GCG	AAUGCACUUAUCCUCGCAAGACACCUAUAGUGAGUCGUAUUA**AUG**GCGGCGGCGGCGGCGGCGGCGGCGGCGGCGGCGGCGGCGGCGGCGGCGGCGGCGGCGGCGGCGGCGGCGGCGGCGGCGGCGGCG**UAA**UGGCUACAGAAGGGC
polyG-GGC	AAUGCACUUAUCCUCGCAAGACACCUAUAGUGAGUCGUAUUA**AUG**GGCGGCGGCGGCGGCGGCGGCGGCGGCGGCGGCGGCGGCGGCGGCGGCGGCGGCGGCGGCGGCGGCGGCGGCGGCGGCGGCGGC**UAA**UGGCUACAGAAGGGC

Supplemental Table 3. qRT-pCR primers (5' to 3').

Nanoluc forward	CAGCCGGCTACAACCTGGAC
Nanoluc reverse	AGCCCATTTTCACCGCTCAG
nGFP forward	TCTTCTTCAAGGACGACGGCAACTAC
nGFP reverse	GTACTCCAGCTTGTGCCCCAGGATGT
