# Supplementary figures and images for: CGG repeats trigger translational frameshifts that generate aggregation-prone chimeric proteins
Source: Nucleic Acids Res. 2022 Jul 29;50(15):8674–89. doi: 10.1093/nar/gkac626 (PMC9410890; doi:10.1093/nar/gkac626)

SF1 (Supplement to F1)

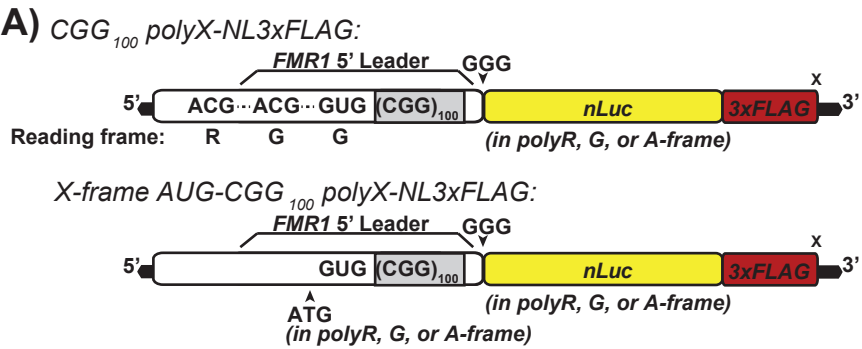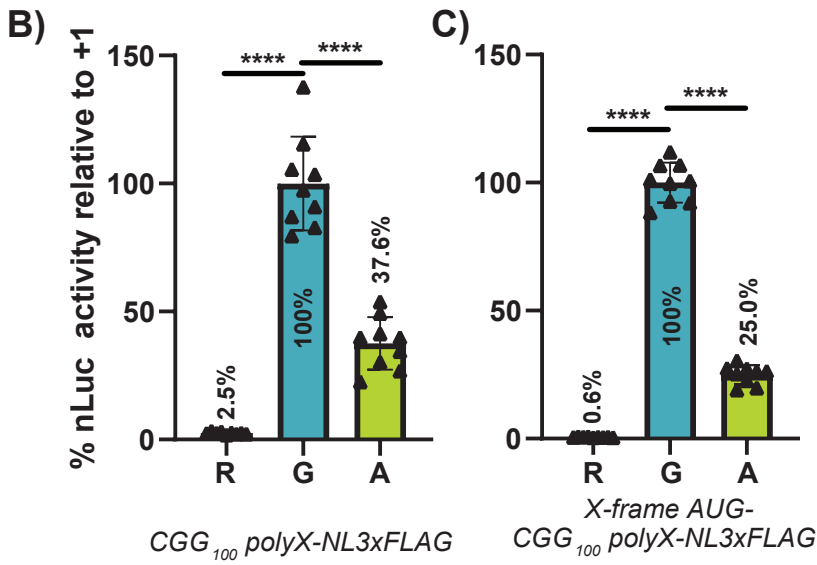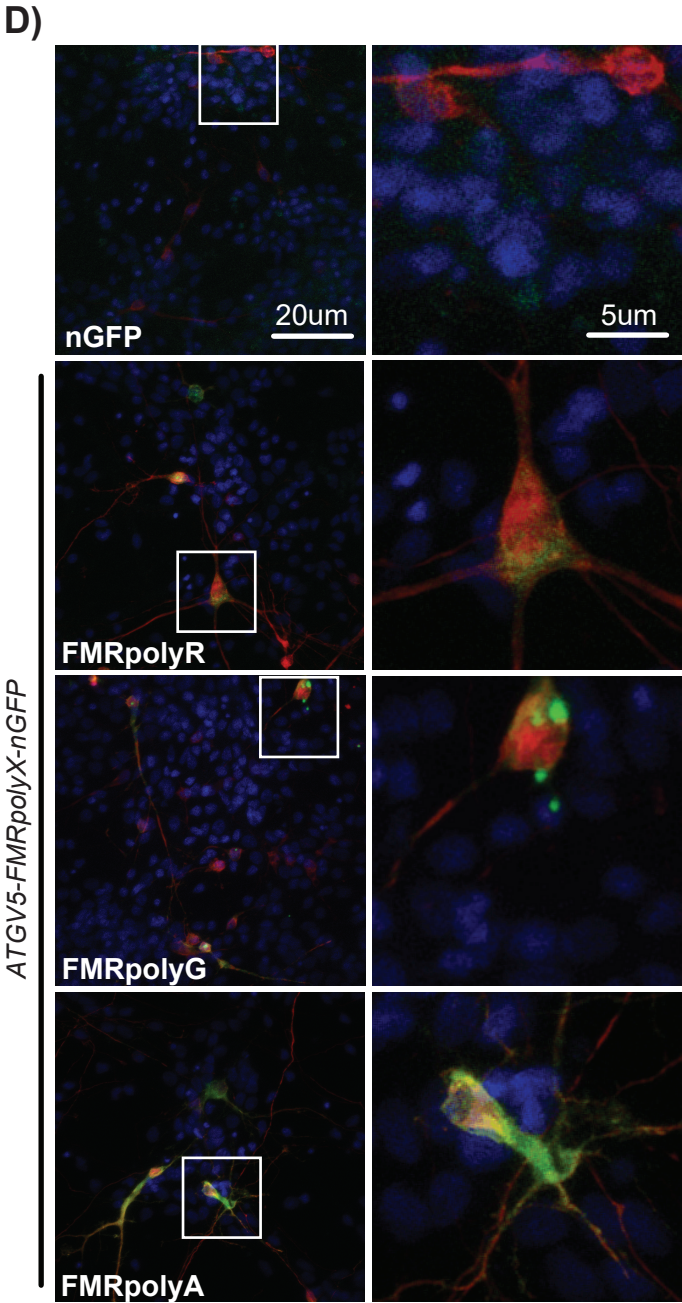

Supplement: gkac626_Supplemental_Files [file gkac626_supplemental_files.zip › SF1.pdf]

# SF2 (Supplement to F1)

A)

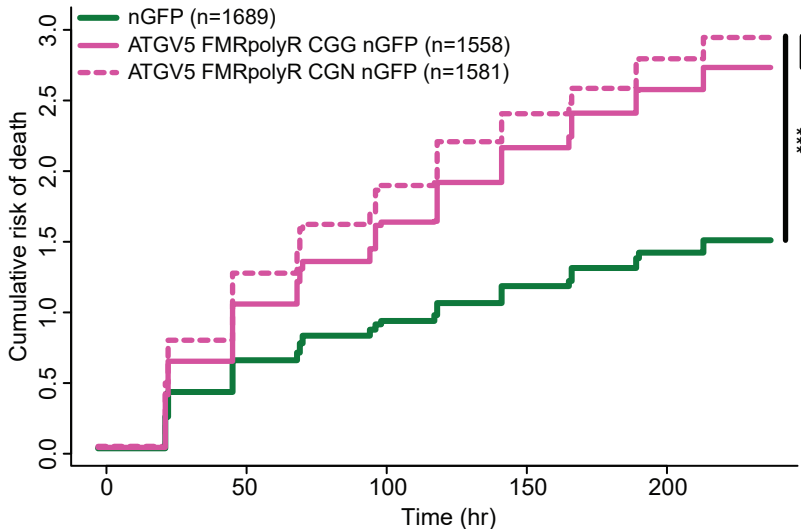

B)

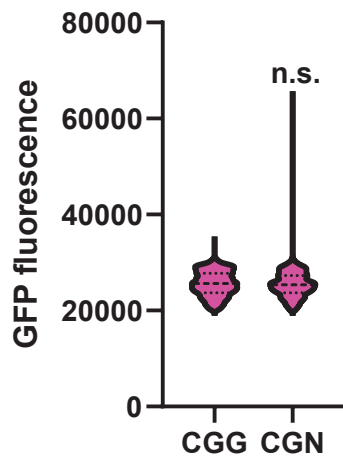

C)

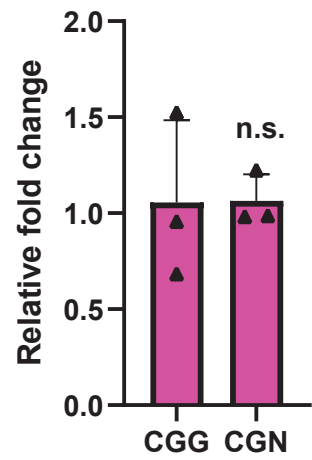

D)

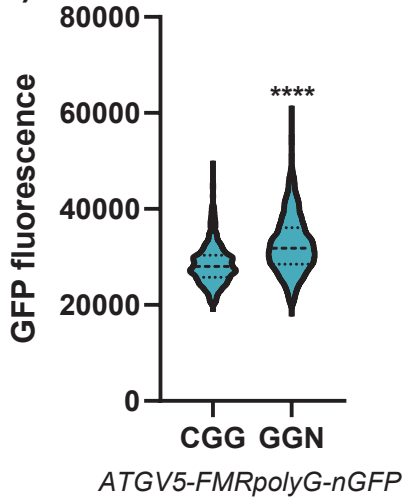

E)

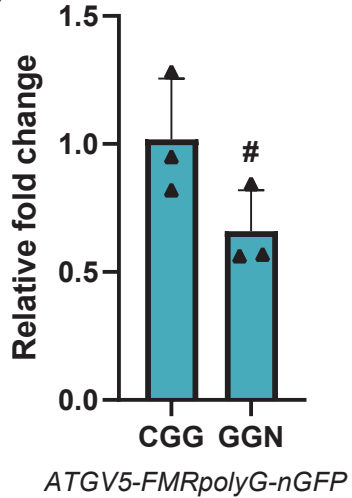

F)

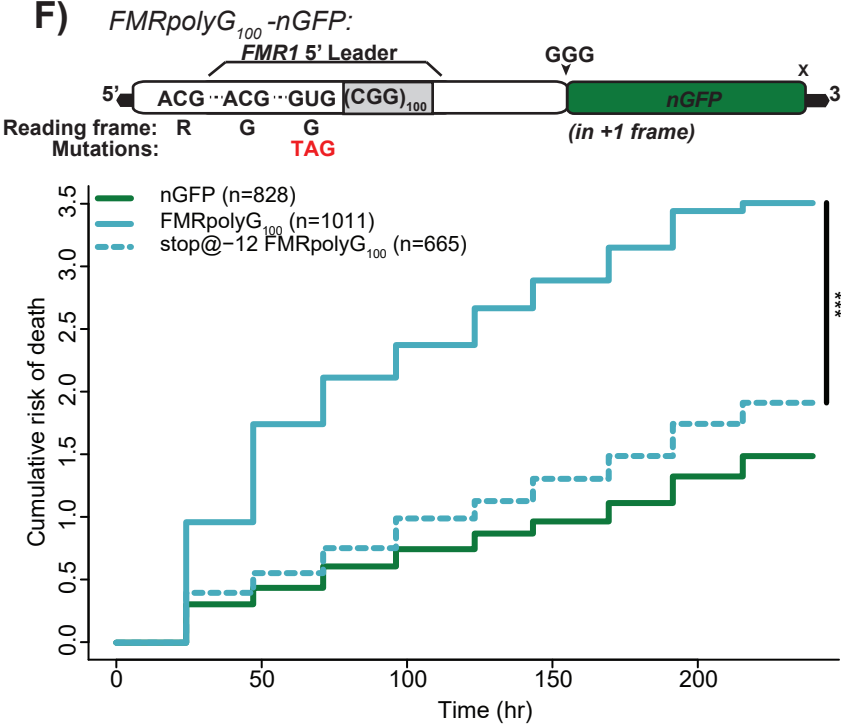

G)

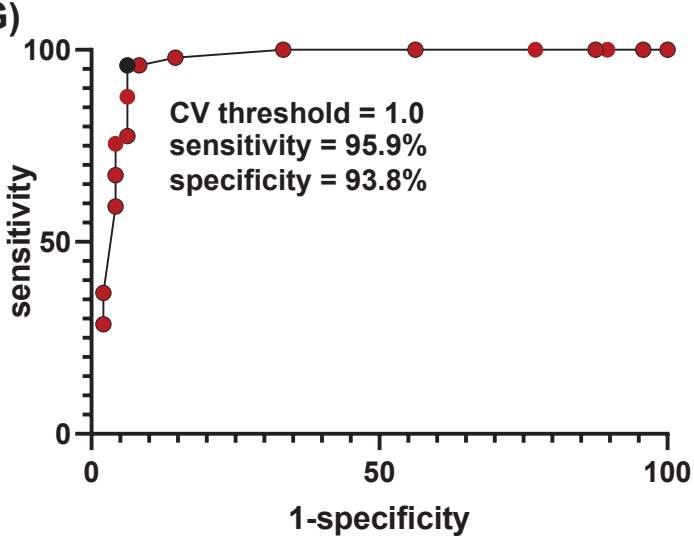

Supplement: gkac626_Supplemental_Files [file gkac626_supplemental_files.zip › SF2.pdf]

# SF3 (Supplement to F2)

A)

## MFold predicted RNA structures

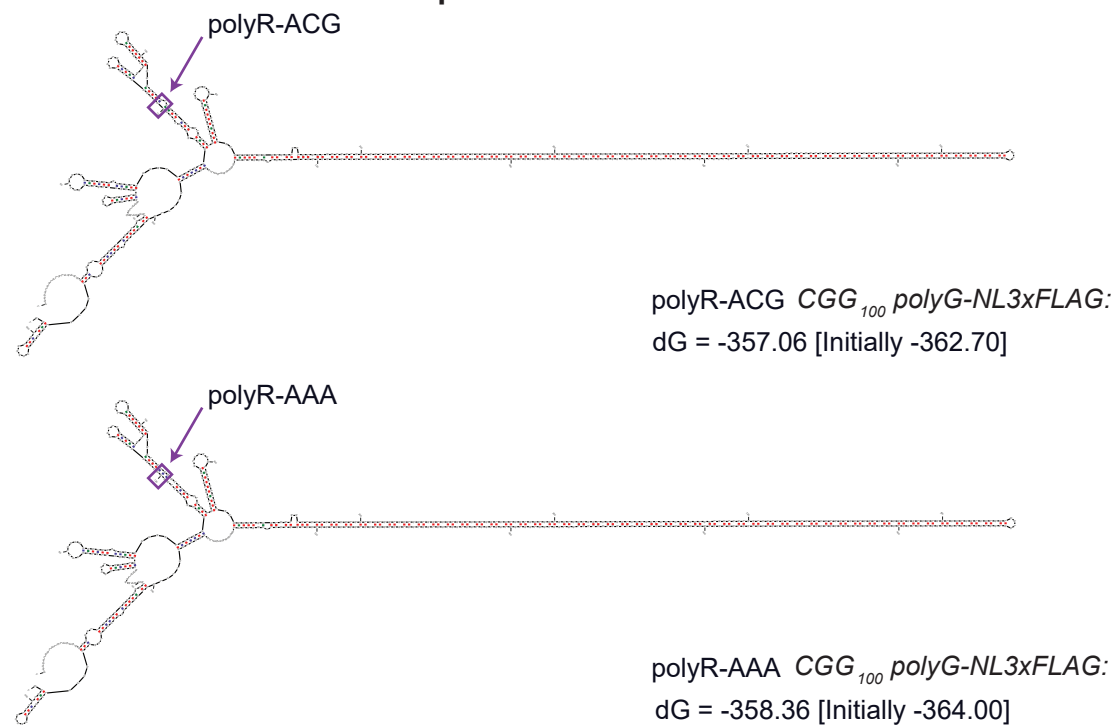

## B) Plasmid Transfection

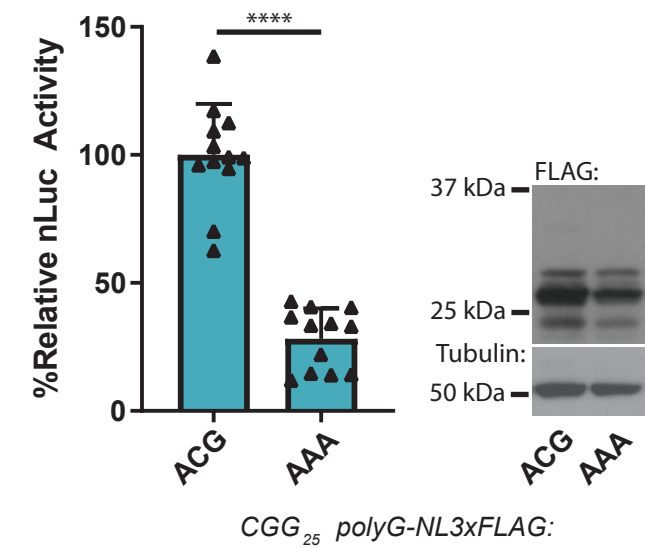

## C) RNA Transfection

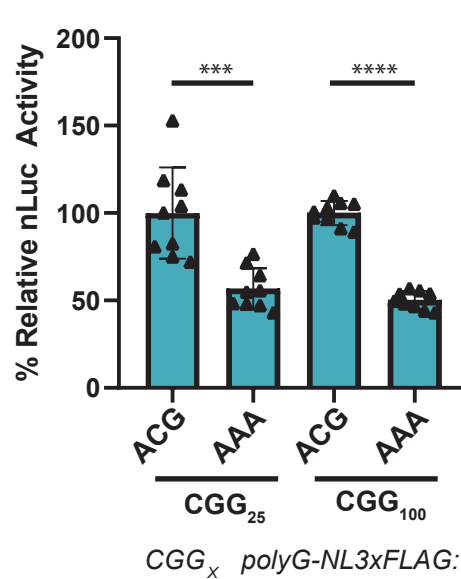

Supplement: gkac626_Supplemental_Files [file gkac626_supplemental_files.zip › SF3.pdf]

SF4 (Supplement to F3)

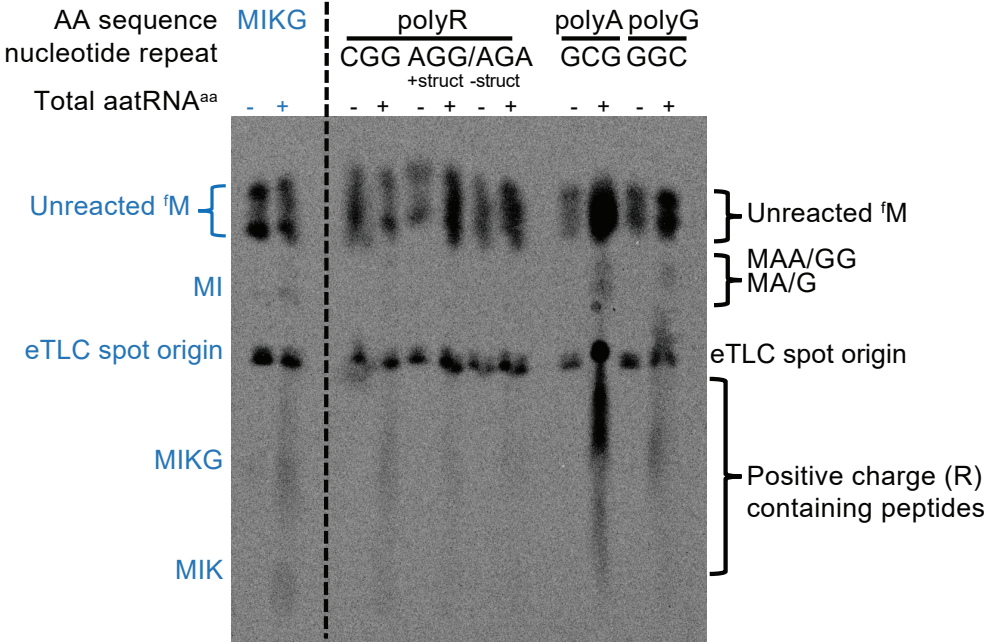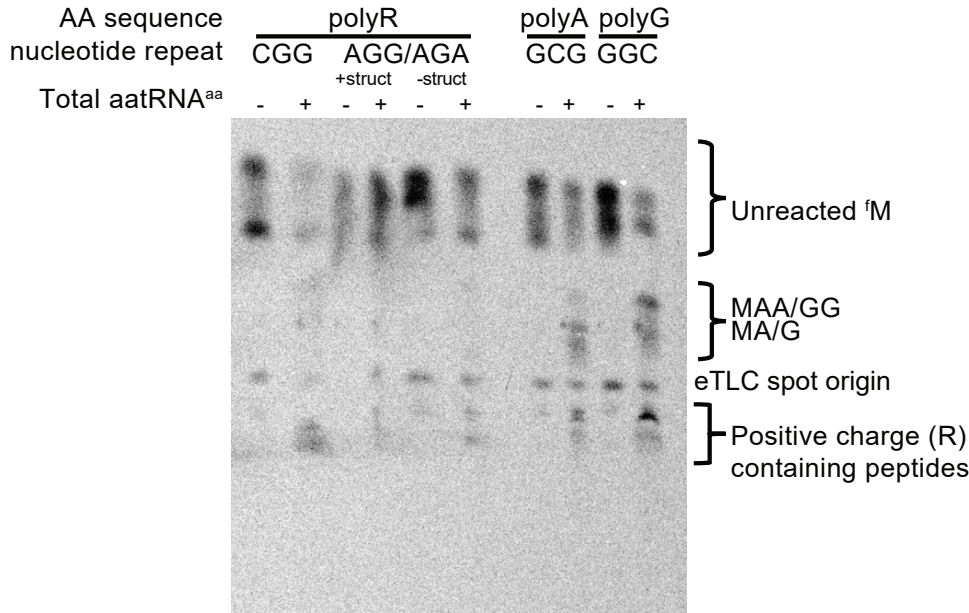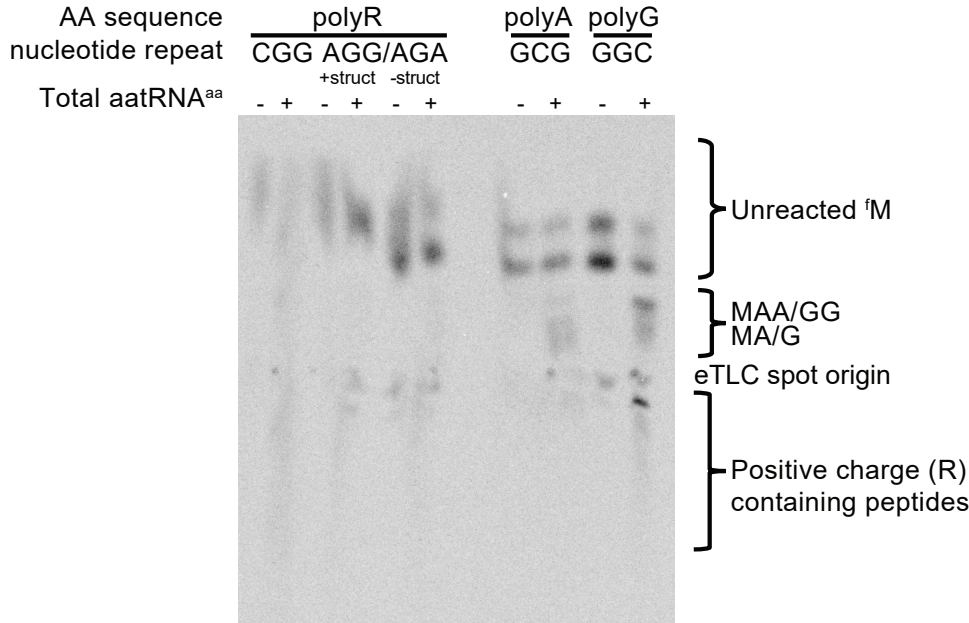

Supplement: gkac626_Supplemental_Files [file gkac626_supplemental_files.zip › SF4.pdf]

SF5 (Supplement to F4)

A) "X-to-Y":

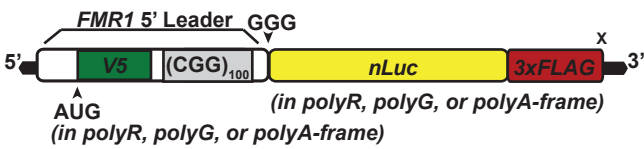

B)

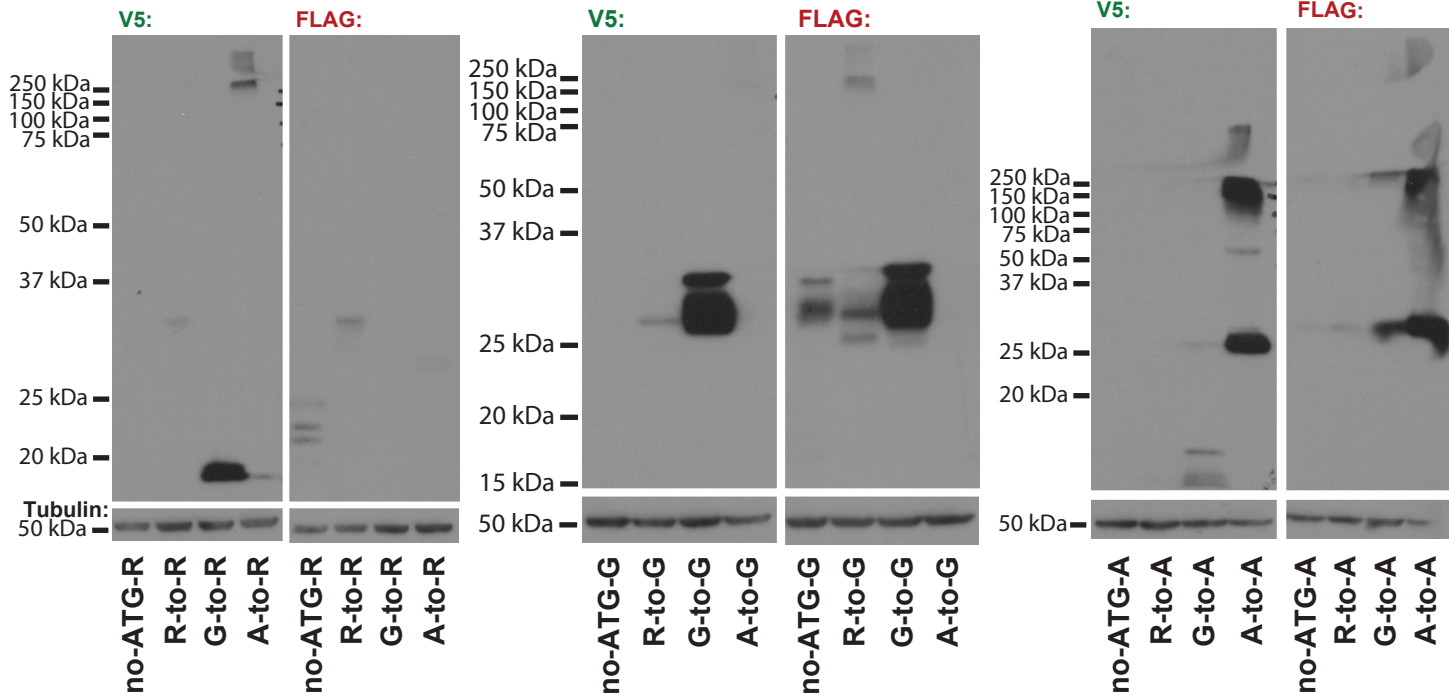

C) "R-to-G":

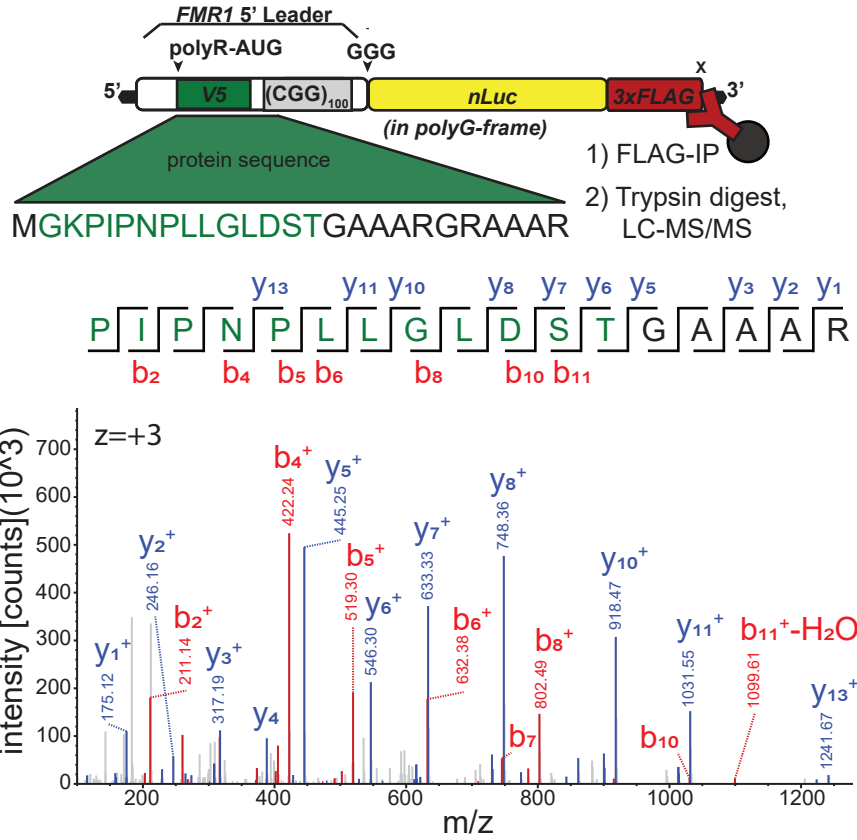

Supplement: gkac626_Supplemental_Files [file gkac626_supplemental_files.zip › SF5.pdf]

SF7 (Supplement to F6)

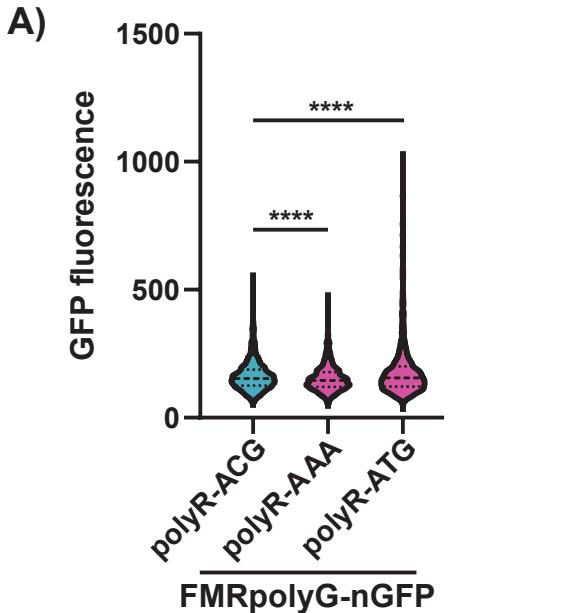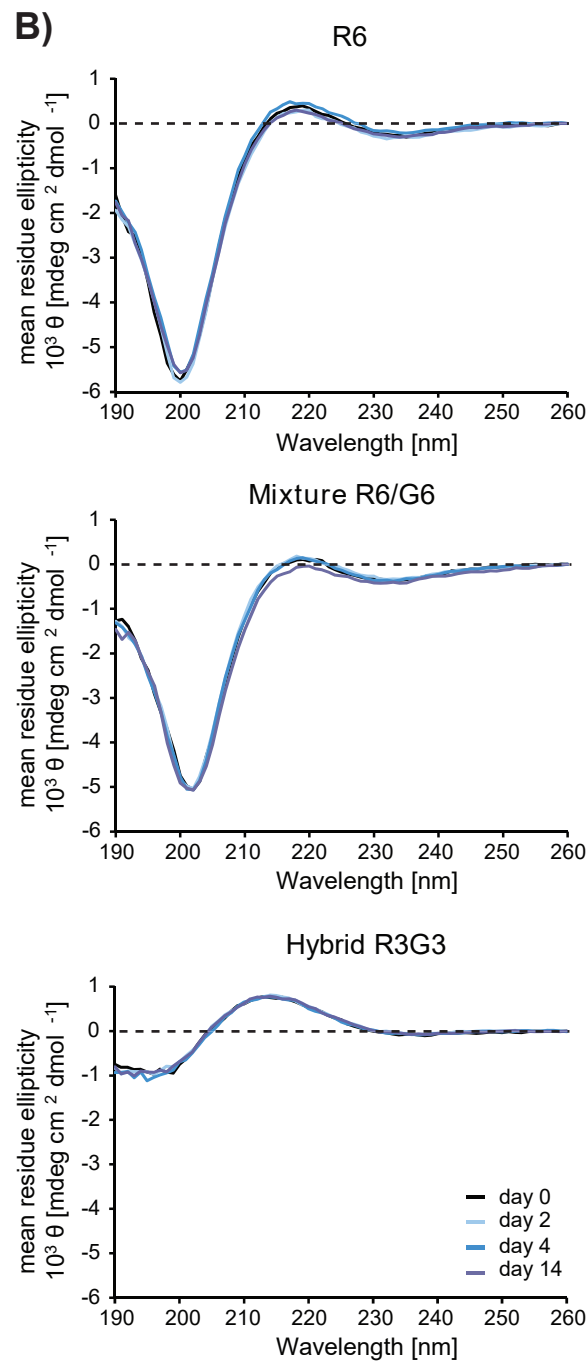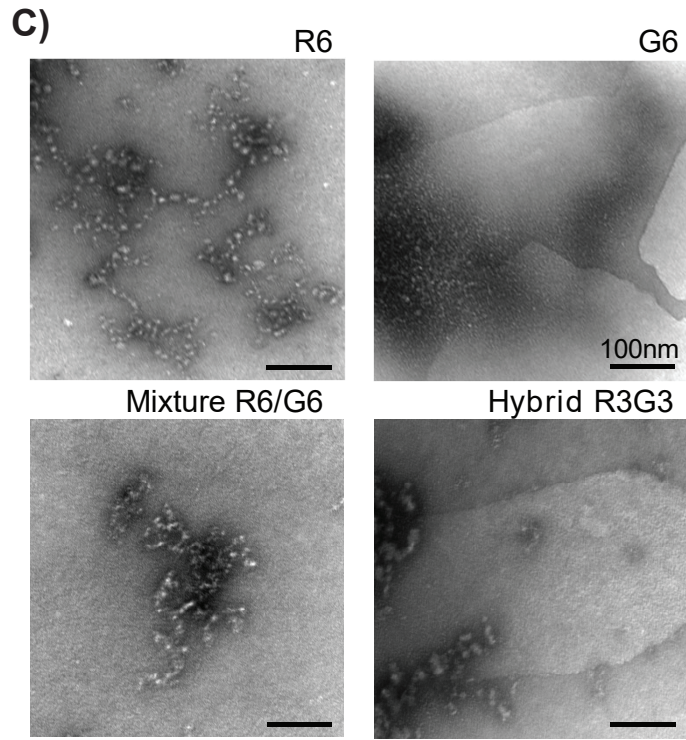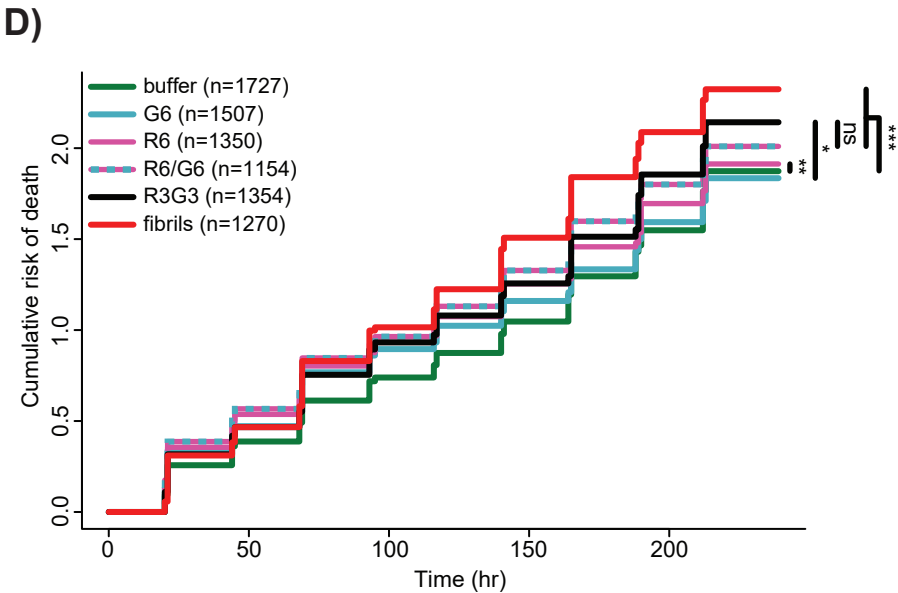

Supplement: gkac626_Supplemental_Files [file gkac626_supplemental_files.zip › SF7.pdf]
